# circCDYL2 promotes trastuzumab resistance via sustaining HER2 downstream signaling in breast cancer

**DOI:** 10.1186/s12943-021-01476-7

**Published:** 2022-01-03

**Authors:** Yun Ling, Gehao Liang, Qun Lin, Xiaolin Fang, Qing Luo, Yinghuan Cen, Maryam Mehrpour, Ahmed Hamai, Zihao Liu, Yu Shi, Juanmei Li, Wanyi Lin, Shijie Jia, Wenqian Yang, Qiang Liu, Erwei Song, Jun Li, Chang Gong

**Affiliations:** 1 Breast Tumor Center, Guangdong Provincial Key Laboratory of Malignant Tumor Epigenetics and Gene Regulation, Sun Yat-sen Memorial Hospital, Sun Yat-sen University, 107 Yanjiang West Road, Guangzhou, 510120 P.R. China; 2 Department of Breast Surgery, the Second Affiliated Hospital, Guangzhou Medical University, Guangzhou, 510260 P.R. China; 3 Department of Breast Oncology, Sun Yat-sen University Cancer Center, Guangzhou, 510080 P.R. China; 4 Institut Necker-Enfants Malades (INEM), Inserm U1151-CNRS UMR 8253, 75993 Paris, France; 5 Université Paris Descartes-Sorbonne Paris Cité, 75993 Paris, France; 6 Department of Biochemistry, Zhongshan School of Medicine, Sun Yat-sen University, Guangzhou, 510080 P.R. China

**Keywords:** circRNAs, Trastuzumab-resistant, HER2 signaling, Breast cancer, GRB7, FAK

## Abstract

**Background:**

Approximate 25% HER2-positive (HER2^+^) breast cancer (BC) patients treated with trastuzumab recurred rapidly. However, the mechanisms underlying trastuzumab resistance remained largely unclear.

**Methods:**

Trastuzumab-resistant associated circRNAs were identified by circRNAs high-throughput screen and qRT-PCR in HER2^+^ breast cancer tissues with different trastuzumab response. The biological roles of trastuzumab-resistant associated circRNAs were detected by cell vitality assay, colony formation assay, Edu assay, patient-derived xenograft (PDX) models and orthotopic animal models. For mechanisms research, the co-immunoprecipitation, Western blot, immunofluorescence, and pull down assays confirmed the relevant mechanisms of circRNA and binding proteins.

**Results:**

We identified a circRNA circCDYL2, which was overexpressed in trastuzumab-resistant patients, which conferred trastuzumab resistance in breast cancer cells in vitro and in vivo. Mechanically, circCDYL2 stabilized GRB7 by preventing its ubiquitination degradation and enhanced its interaction with FAK, which thus sustained the activities of downstream AKT and ERK1/2. Trastuzumab-resistance of HER2^+^ BC cells with high circCDYL2 could be reversed by FAK or GRB7 inhibitor. Clinically, HER2^+^ BC patients with high levels of circCDYL2 developed rapid recurrence and had shorter disease-free survival (DFS) and overall survival (OS) following anti-HER2 therapy compared to those with low circCDYL2.

**Conclusions:**

circCDYL2-GRB7-FAK complex plays a critical role in maintaining HER2 signaling, which contributes to trastuzumab resistance and circCDYL2 is a potential biomarker for trastuzumab-resistance in HER2^+^ BC patients.

**Supplementary Information:**

The online version contains supplementary material available at 10.1186/s12943-021-01476-7.

## Background

Breast cancer (BC) is the most common malignant tumor globally with a 0.3% increasing rate per year [1]. Among these BC patients, 15–20% exhibits amplification/overexpression of human epidermal growth factor receptor 2 (HER2), subtyped as HER2-positive (HER2^+^) breast cancer [2]. Trastuzumab, a recombinant mAb binding to the extracellular domain of HER2, is the first-line recommended drug for HER2^+^ BC patients and has been proved to prolong survival of HER2^+^ BC patients [3]. Although the prognosis of HER2^+^ patients is largely favourable with the use of trastuzumab, 25–40% HER2^+^ patients still suffer recurrence and metastasis due to trastuzumab resistance [2, 4]. Unfortunately, HER2^+^ BC patients with intrinsic trastuzumab-resistance have short survival-benefit from alternative anti-HER2 drugs, which accounts for a high rate of death [5]. Therefore, it is of great significance to elucidate the mechanism of trastuzumab resistance in HER2^+^ BC.

Trastuzumab treatment could inhibit dimerization of HER2 protein and downstream signaling, decrease DNA repair, increase apoptosis and impede angiogenesis in HER2+ breast cancer [6, 7]. In recent years, compelling evidence has been provided that aberrant activation of PI3K/AKT or RAS/ERK signaling pathway, the HER2 downstream signaling pathway, play vital roles in trastuzumab resistance [8, 9]. Therefore, multiple PI3K inhibitors and MEK inhibitors have been employed and showed significant effect on increasing of trastuzumab sensitivity or reversing trastuzumab resistance of HER2+ BC patients [10–13]. However, aberrant activation of PI3K/AKT or RAS/ERK usually simultaneously exist and cooperate together to contribute to trastuzumab resistance, which results in inhibition of single pathway is not always efficient for trastuzumab-resistant patients. Numerous researches show that multiple pathway-targeted drugs combination has a powerful killing effect for HER2^+^ BC cells. In addition, many drugs like special small molecule compounds, such as BEZ235 (a small molecule compound that targets both mTOR and PI3K), are designed to target multiple signaling pathways to reverse trastuzumab resistance of HER2^+^ BC patients. However, due to lack of sufficient effectiveness and safety data, clinical applications of these new drugs or multi-drug combination for trastuzumab-resistant patients are not clear and most of them stay at the preclinical stage [14]. Therefore, it is of great importance to find out new molecules that trigger activation of multiple trastuzumab-resistance-associated pathways.

Circular RNAs (circRNAs) are a group of covalently closed RNA molecules without 3′ or 5′ ends [15, 16]. CircRNAs show disease-specific and disease-progression-specific characteristics [17, 18], and exhibit powerful biological functions in regulating malignant behaviors such as proliferation, migration, autophagy, drug resistance etc. of breast cancer cells [19, 20]. As reported, circRNAs regulate biological functions through multiple mechanisms, such as microRNA sponges [19, 20], proteins translation [21] or binding to RNA-binding proteins [22]. Some circRNAs are indicated to activate multiple signaling pathways that may cause trastuzumab resistance. For example, circMAT2B promotes the activation of both PI3K/AKT and Raf/MEK/ERK pathways in cardiomyocytes [23]. However, there is no direct evidence nowadays to reveal the role of circRNAs in trastuzumab resistance of HER2^+^ BC patients. Here, through high-throughput screening for clinical samples and patient-derived xenograft (PDX) models, we investigated whether and how circRNAs may sustain the activity of HER2 signaling pathway, consequently resulting in trastuzumab resistance.

## Material and methods

### Patient samples and clinical database

In our study, three independent cohorts with HER2^+^-breast cancer patients were enrolled at Sun Yat-sen Memorial Hospital (SYSMH). The enrolled patients were first diagnosed without any distant metastasis between 1st January 2010 and 31th December 2017. All of these patients received standard clinical therapy according to the National Comprehensive Cancer Network (NCCN) guide line. Disease free-survival (DFS) was defined as the interval between the date of diagnosis and the first local or distant disease recurrence, or the last follow-up with relevant event. Overall survival (OS) was defined as the interval between the date of diagnosis and death by breast cancer. Fresh BC patient tissues in cohort 1 (divided into trastuzumab-resistant group and trastuzumab-sensitive group after anti-HER2 therapy) were collected for circRNAs sequencing (*n* = 10). Cohort 2 (divided into trastuzumab-resistant group and trastuzumab-sensitive group) were selected for qRT-PCR detection (*n* = 71). Paraffin-embedded breast cancer patient tissues samples in cohort 3 (*n* = 127) were used for immunohistochemical staining (IHC) and in situ hybridization (ISH). corhort 2 included 39 patients from cohort 3. All work was approved by Sun Yat-sen Memorial Hospital Ethics Committee (SYSEC-KY-KS-2019-137).

### In situ hybridization (ISH)

The protocol of ISH performed on paraffin sections of breast cancer tissues or animal tumors was taken from previous literature. Briefly, after dewaxing and rehydration, the sections were digested with pepsin, and then hybridized with the digoxin-labelled probe (Synbio-Tech, Guangzhou, China) overnight at 37 °C. Next, the sections were incubated with anti-digoxin antibody overnight at 4 °C. The intensity and proportion at whole section was recorded on a scale of 0 (no staining), 1 (light purple), 2 (purple blue), and 3 (dark purple). Total score of expression = ∑proportion * intensity score.

### In vivo animal experiment

We kept the tissues in PRI DMEM with 10% foetal bovine serum and 1% penicillin/streptomycin, cut them into 1 × 1 × 1 mm^3^ pieces and washed the pieces with fresh PRI DMEM twice. Then, we buried the tissues subcutaneously in NOD/SCID mouse fat pad. When the xenografted tumor tissues grow to 1–2 cm^3^, we followed the protocols as we aforementioned to harvest the tissues and transplanted them into Balb/c-nude mice as subsequent generations. In the fourth generation, we injected the cholesterol-conjugated circCDYL2 siRNA and cholesterol-conjugated negative control siRNA into tumor tissues continuously from day 0 to day 25 (1.0 nmol/20 g, diluted in distilled water) and then the tumor tissues were harvested on day 44 for further analysis [24, 25]. For biosafety evaluation, the weight of mice was recorded every 2 days for 4 weeks, and we did not observe statistic difference on the weight of mice with administration of distilled water, cholesterol-conjugated negative control siRNA and cholesterol-conjugated circCDYL2 siRNA.

Four-week-old female Balb/c nude mice were purchased from Nanjing Biomedical Research Institute of Nanjing University (Nanjing, China) and housed under standard conditions at the animal care facility at Forevergen Medical Corporation (Guangzhou, China). SK-BR-3-R cells transduced with sh-circCDYL2, sh-NC, and P-SK-BR-3 cells transduced with empty vector (ev) or circCDYL2 (p-circ) were implanted into the left fourth mammary fat pads of Balb/c nude mice. All of them were also injected with trastuzumab (10 mg/kg), and other nude mice group were implanted with sh-NC but without trastuzumab. When the tumors of NC group had grown to 400 mm^3^, the nude mice were sacrificed and then the tumors were extracted for in situ hybridization (ISH) and immunohistochemistry (IHC). The tumor size was measured every 5 days by using Vernier calliper. Tumor volume = length x width^2^.

### Fluorescence in situ hybridization (FISH)

Cy3-labeled oligonucleotide probe for circCDYL2 (Synbio-Tech, Guangzhou, China) was used for FISH (sequence shown in Table S1). Cells were seeded in a confocal dish and incubated with pre-hybridization solution at 37 °C for 30 mins. Twenty micrometre probes were added to the dish and hybridized overnight. After washing (4× SSC (saline sodium citrate) in 0.1% Tween-20 for three times, 2 × SSC for once, and 1× SSC for once), the cells were stained with DAPI for 15 mins at RT. Finally, images were observed under a confocal microscope.

### CircRNA pull-down

CircRNA pull-down was performed using biotinylated circCDYL2 probe and a negative control (NC) probe (Sangon Biotech, China), according to previous literatures [26, 27]. In brief, SK-BR-3 cells were cross-linked by 3% formaldehyde for 30 min and lysed by co-IP buffer at room temperature (RT). Then circCDYL2 and NC biotinylated probes were added to the supernatant and incubated overnight at 37 °C. The next day, the samples were co-incubated with C1 streptavidin magnetic beads (65,001, Invitrogen, USA) at 37 °C for 30 min. Finally, total RNA mixture was extracted to detect circCDYL2 by qRT-PCR. Proteins were separated by SDS-PAGE and executed by sliver staining. Mass spectrum analysis was then performed to find potential binding protein.

### RNA immunoprecipitation (RIP)

The RIP assay was performed according to RNA-Binding Protein Immunoprecipitation Kit manual (17–700, Millipore, USA). In brief, SK-BR-3 cells were washed by cool PBS twice and lysed by co-IP buffer. The lysates were first co-incubated with anti-GRB7 antibody (376,069, Santa Cruz, USA, 1:1000), or negative control IgG (2,822,115, Millipore, USA, 1:1000) and anti-FAK antibody (13,009, Cell Signaling Technology, USA, 1:1000) overnight at 4 °C. Subsequently, the protein A/G beads were co-incubated with immunocomplex mixture for 2 h in 4 °C. Total RNA of the immunocomplex was extracted by TRIzol reagent (Invitrogen, Carlsbad, USA), then the circCDYL2 expression was detected by qRT-PCR.

### Co-Immunoprecipitation (co-IP)

The Co-IP assay was performed according to previous literature [28]. SK-BR-3 cells were washed by cool PBS twice and lysed by co-IP buffer. The lysates were first co-incubated with anti-GRB7 antibody (376,069, Santa Cruz, USA, 1:1000) or anti-FAK antibody (13,009, Cell Signaling Technology, USA, 1:1000) overnight at 4 °C. Subsequently, the protein A/G beads were co-incubated with mixture for 2 h in 4 °C. The beads were washed with PBS, then resuspended by 1 × SDS-PAGE Loading Buffer. Total protein of the immunocomplex was subjected to Western Blot with antibodies against the other protein. For detecting the ubiquitination of GRB7, cells were treated with MG132 (474790, Sigma-Aldrich, USA).

### Statistical analysis

The experimental data was analyzed by using Student’s t-test or one-way ANOVA in GraphPad Prism 5 software. Kaplan-Meier plots and Log-rank tests were performed for survival analysis. The correlations between different molecules were calculated using Pearson’s correlation coefficients. *P* < 0.05 was considered statistically significant.

### Additional material and methods

Additional materials and methods can be found in supplementary Information.

## Results

### Clinical significance and characterization of circCDYL2

**Fig. 1:**
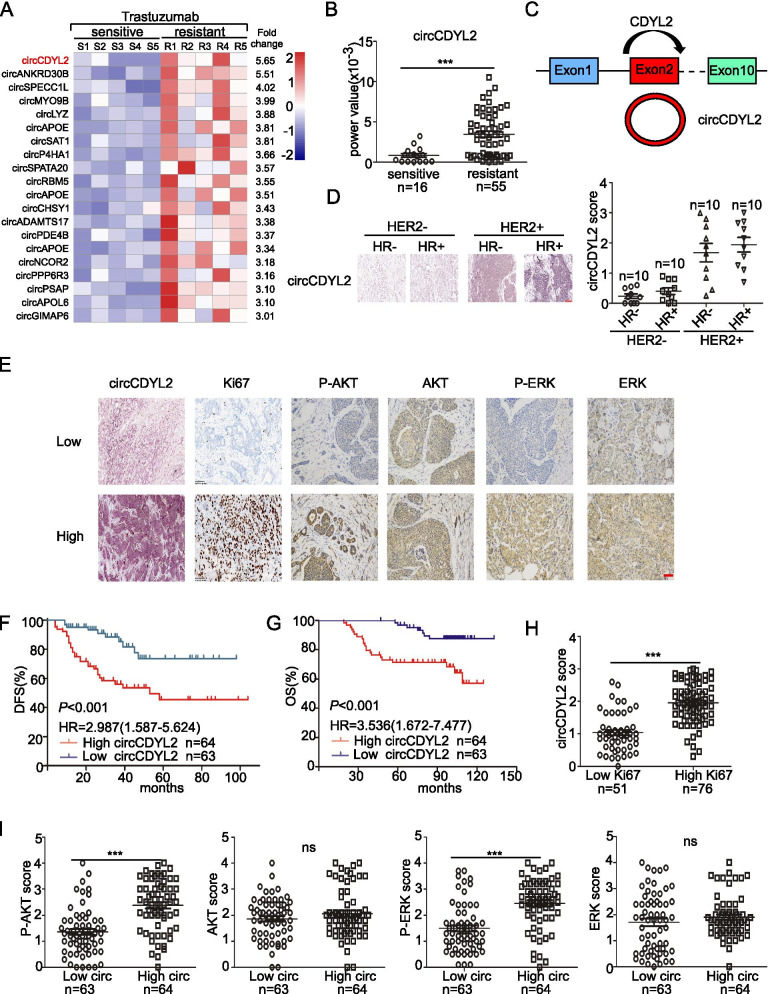
Identification of Trastuzumab-resistant associated circRNA (circCDYL2) in breast cancer after anti-HER2 therapy. **A** circRNA expression profile in breast cancer patients with Trastuzumab sensitive and resistant by circRNAs deep sequencing. R, trastuzumab-resistant patient & S, trastuzumab-sensitive patient. **B** Quantitative analysis of circCDYL2 expression in HER2^+^ cancer tissues with trastuzumab sensitive (*n* = 16) and trastuzumab resistant (*n* = 55) by qRT-PCR. **C** The origin of circCDYL2. **D** The expression of circCDYL2 in different breast cancer patient tissues by ISH**.** HR, hormone receptor. Scale bar, 100 μm. **E** Representative images of circCDYL2 by ISH and Ki67, P-AKT, AKT, P-ERK and ERK by IHC. Scale bar, 50 μm. **F** Kaplan-Meier analysis of the correlation between circCDYL2 expression and disease-free survival (DFS). All of the 127 HER2^+^ BC patients were received standard trastuzumab therapy. **G** Kaplan-Meier analysis of the correlation between circCDYL2 expression and overall survival (OS). **H** The relationship of expression between circCDYL2 and Ki67. **I** The relationship of expression between circCDYL2 and P-AKT, AKT, P-ERK and ERK. All experiments were repeated at least 3 times. *P < 0.05, **P < 0.01, ***P < 0.005. Error bars indicate Standard Error of Mean (S.E.M)

Due to the poor prognosis of patients with trastuzumab resistance, exploring molecular markers that can predict the efficacy of trastuzumab and targets for the treatment of trastuzumab resistance are important. CircRNA deep RNA sequencing was performed in cohort 1, which included five patients were sensitive to trastuzumab and five patients were resistant to trastuzumab after anti-HER2 therapy (Fig. 1A, Table S2). The CircRNA deep RNA sequencing analysis revealed that a total of 3498 circRNAs, including 3356 upregulated circRNAs and 138 downregulated circRNAs, were dysregulated in the trastuzumab-sensitive breast cancer tissues compared to the trastuzumab-resistant tissues. The most upregulated twenty circRNA candidates were selected according to following standards: fold changes (resistant subgroup VS. sensitive subgroup) > 3 and junction reads per million mapped reads (RPM) of sensitive subgroup > 2 (Table S3). Then we quantified the expression level of top eight circRNAs in cohort 2 that included sixteen HER2^+^ BC patients who were sensitive to trastuzumab HER2^+^ BC patients and fifty-five resistant to trastuzumab. We found that circCDYL2, circSPECC1L and circSAT1 were significantly upregulated in trastuzumab-resistant subgroup (Fig. 1B, Fig. S1A, Table S4). Firstly, we established two trastuzumab-resistant cell lines including SK-BR-3 and BT474. The real-time PCR analysis showed that only circCDYL2 (hsa_circ_0004087, chr16:80718434–80,719,026), which was derived from exon 2 of Chromodomain Y Like 2 gene (CDYL2), was highly expressed in trastuzumab-resistant cell lines than parental cell lines (Fig. 1C, Fig. S1B, C). Then, we tested the expression level of circCDYL2 in different BC subtyping tissues, and found that circCDYL2 was higher expression in HER2 positive subtype than HER2 negative subtype (Fig. 1D). Clinical data from 127 cases of HER2^+^ early BC patients who recieved trastuzumab treatment indicated that the high-circCDYL2-expressing HER2^+^-BC tumors were significantly associated with larger tumour size, increased lymphatic metastasis and higher Ki67 index (Table 1). More importantly, HER2^+^ patients with high circCDYL2 had higher recurrence rate after trastuzumab treatment. Further survival analysis suggested that patients with high circCDYL2 expression had shorter disease-free survival (DFS) (HR = 2.987, 95%CI = 1.587–5.624, *P* < 0.001), and shorter over-survival (OS) (HR = 3.536, 95%CI = 1.672–7.477, *P* < 0.001) than those with lowcircCDYL2 level (Fig. 1E-G). To explore the mechanism of trastuzumab-resistant, we also identified the PI3K/AKT and MERK/ERK pathways. We found that tumors with higher circCDYL2 had higher expression of Ki-67, P-AKT and P-ERK (Fig. 1H-I). Clinical data above revealed that circCDYL2 expression was associated with trastuzumab resistance in HER2^+^ BC patients, and related with phosphorylation of AKT and ERK.

To further prove the circular structure of circCDYL2, we designed divergent primers and convergent primers to amplify circCDYL2 and linear CDYL2 respectively (Fig. S1D). As shown in Fig. S1E, circCDYL2 could only be amplified by divergent primers in cDNA, not gDNA and the sequence of qRT-PCR products of circCDYL2 by divergent primers matched the junction sequence of circCDYL2 in the circBase database. Furthermore, we found that circCDYL2 was more stable than CDYL2 mRNA after treatment by RNase R (Fig. S1F). circCDYL2 was also more stable than linear CDYL2 in living cells after inhibiting transcription of SK-BR-3 cells by actinomycin D (Act D, a transcription inhibitor) (Fig. S1G). Meanwhile, circCDYL2 could only be reversed by random primer, while linear CDYL2could be reversed by both random primer and oligo dT primers (Fig. S1H). Taken together, all results above indicated circCDYL2 was a circular RNA.

### circCDYL2 promotes trastuzumab resistance on PDX models and HER2^+^ cells

**Fig. 2:**
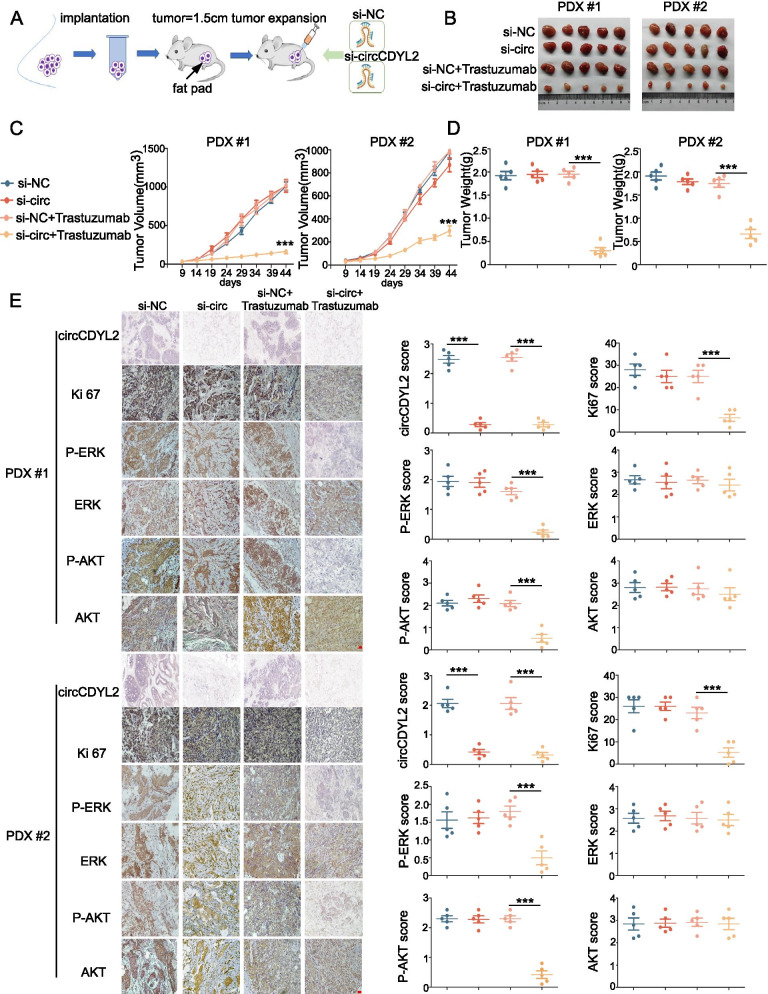
circCDYL2 promotes drug resistant to trastuzumab in the PDX model. **A** The establishment of xenograft model derived from trastuzumab-resistant patient tissues. **B** Tumor derived from trastuzumab-resistant patient was measured in Balb/c nude mice. **C**, **D** Tumor volume (**C**) and tumor weight (**D**) was measured after tumor excision. **E** IHC staining of Ki-67, P-ERK, ERK, P-AKT and AKT and ISH detection of circCDYL2 in xenograft tumors. Scale bar, 50 μm. *n* = 5 animals per group

To explore the mechanism underlying circCDYL2 promoted trastuzumab resistant on patient-derived xenograft (PDX) models, we established two PDX models using HER2^+^ patient tissues who were resistant to trastuzumab (Fig. 2A, Table S5). After continuous intratumoral injection of cholesterol-conjugated circCDYL2 siRNAs for 4 weeks, we found that si-circCDYL2 group had more sensitive about trastuzumab than the si-NC group (Fig. 2B-D). We performed ISH and IHC staining on two cases of PDX models to determine the level of circCDYL2, Ki67, AKT and ERK. The expression of circCDYL2 and Ki-67 were significantly decreased in si-circCDYL2 group compared to si-NC group. Moreover, the level of P-AKT and P-ERK were markedly decreased by circCDYL2 knockdown (Fig. 2E). All these results indicate that circCDYL2 contributes to trastuzumab resistance of HER2^+^-breast cancer on PDX models.

**Fig. 3:**
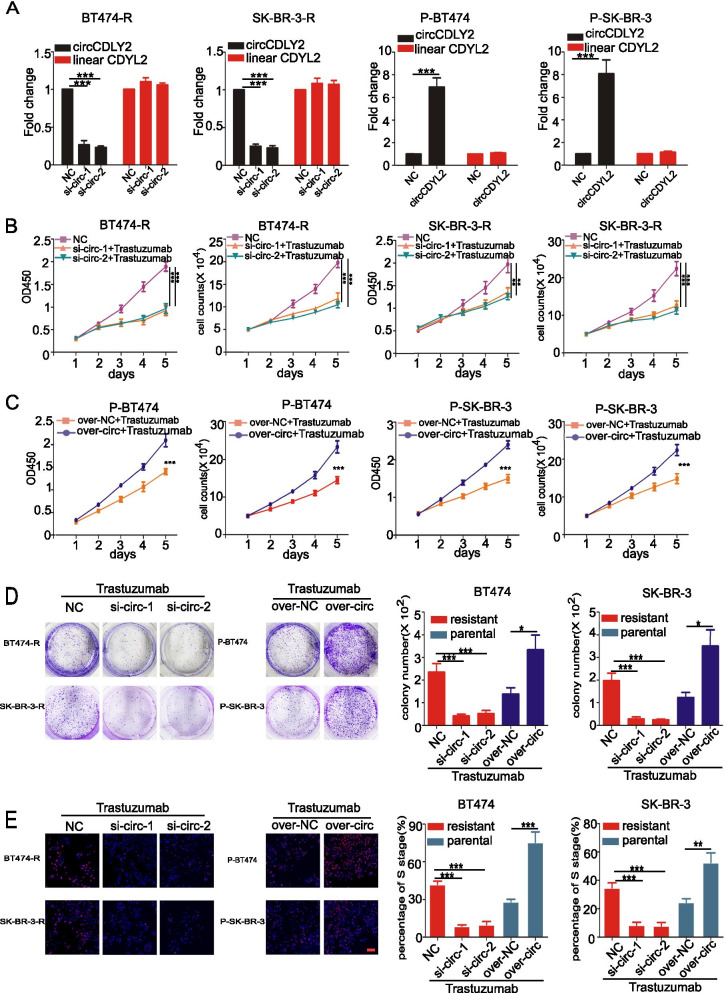
circCDYL2 promotes trastuzumab-resistant of HER2^+^ BC cells. **A** qRT-PCR analysis of circCDYL2 and linear CDYL2 in both BT474-R, SK-BR-3-R, P-BT474 and P-SK-BR-3 cells after circCDYL2 silencing or over-expressing by circCDYL2 specific siRNAand over-expressing plasmid. **B-D** The cell viability of BC cells after treatments with circCDYL2 siRNA or over-expressing circCDYL2 plasmid or trastuzumab, as detected by CCK8 assay (**B**), cell viability assay (**C**) and colony formation (**D**). **E** circCDYL2 promotes the trastuzumab resistant of BC cells shown by the EdU assay. 2 μg/ml Trastuzumab was added to cells every day. Scale bar, 50 μm. All experiments were repeated at least 3 times. *P < 0.05, **P < 0.01, ***P < 0.005. Error bars indicate S.E.M.

To further investigate whether circCDYL2 contributed to trastuzumab resistance of HER2^+^-BC, we designed relative in vitro experiments. Then, we stably knocked down circCDYL2 using siRNA in BT474-R (trastuzumab-resistant BT474) and SK-BR-3-R (trastuzumab-resistant SK-BR-3) cell lines, and over-expressed circCDYL2 in P-BT474 (parental BT474) and P-SK-BR-3 (parental SK-BR-3) cells by plasmids (Fig. 3A). As shown in Fig. 3A, either silencing or overexpressing circCDYL2 had no effect on linear CDYL2 expression. CCK8 assay indicated silencing circCDYL2 slightly inhibited cell proliferation. However, in the presence of trastuzumab treatment, knock-down circCDYL2 observably inhibited cell viability in both BT474-R and SK-BR-3-R, while over-expressing circCDYL2 obviously increased viability in both P-BT474 and P-SK-BR-3 cell (Fig. 3B, C). Similar results were also found by colony formation and EdU assay (Fig. 3D, E). Meanwhile, knocking down circCDYL2 only slightly impaired the migratory ability of BT474 and SK-BR-3 cells (Fig. S2A,) and had no significant effect on apoptosis (Fig. S2B). As shown in Fig. S2C, siRNAs-mediated downregulation of circCDYL2 in JIMT-1 cells had no effect on linear CDYL2 expression. Furthermore, multiple functional assays, including colony formation, EdU, CCK8 and cell vitality assays, revealed that silencing circCDYL2 significantly abrogated the trastuzumab-resistant capability of JIMT-1 cells (Fig. S2D-G). Moreover, we found that the downregulation of circCDYL2 dramatically reduced the expression of phosphorylated-AKT and -ERK1/2 (Fig. S2H). Therefore, these results provided further evidence that circCDYL2 overexpression resulted in trastuzumab resistance via sustaining HER2 downstream signaling in breast cancer. Moreover, we found that overexpressing circCDYL2 could not influence the proliferation of HER2 negative cells (MDA-MB-231 and MCF-7) (Fig. S2I-K). In vitro experiments suggest that circCDYL2 is related with trastuzumab resistant in HER2^+^ BC cells.

### circCDYL2 interacts with GRB7 protein

**Fig. 4:**
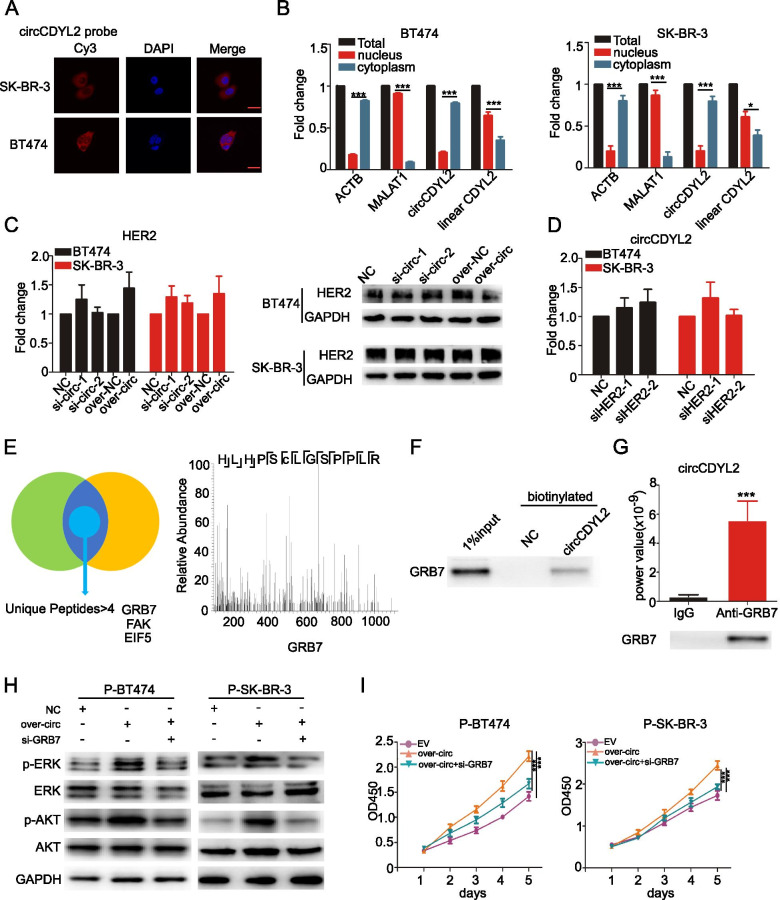
circCDYL2 interacts with GRB7 protein in HER2^+^ BC cells. **A** Location of circCDYL2 in both BT474 and SK-BR-3 cells by FISH detection. Scale bar 50 μm. **B** qRT-PCR analysis of circCDYL2 in the cytoplasm and nuclear separated by PARIS kit. **C** qRT-PCR and Western Blot analysis of HER2 in BT474 and SK-BR-3 cells after silencing and over-expressing circCDYL2. **D** qRT-PCR analysis of circCDYL2 in both BT474 and SK-BR-3 after silencing HER2. **E** Mass spectrum of GRB7 protein. **F** Biotinylated circCDYL2 probe pull down results for GRB7 protein by Western Blot. **G** qRT-PCR analysis of circCDYL2 in RNA sample after RIP assay by anti-GRB7antibody. **H** Total and phosphorylased of AKT and ERK1/2 expression in both P-BT474 and P-SK-BR-3 cells, as detected by Western Blot after co-transfection with circCDYL2-overexpressing plasmid or GRB7 siRNA with Trastuzumab. **I** The proliferation of P-BT474 and P-SK-BR-3 cells after co-transfection circCDYL2-overexpressing plasmid or GRB7 siRNA with trastuzumab, as detected by CCK8 assay

Next, we explored how circCDYL2 induced trastuzumab resistance. We firstly examined the location of circCDYL2. Both fluorescence in situ hybridization (FISH) (Fig. 4A) and nuclear-cytoplasm separation experiments (Fig. 4B) showed that circCDYL2 was mainly located in cytoplasm. Since circCDYL2 was overexpressed in HER2^+^ BC cells, we investigated the relationship between circCDYL2 and HER2 expression. As shown in Fig. 4C-D, neither silencing nor overexpressing circCDYL2 had an effect on HER2 expression at both RNA and protein level, and dysregulation of HER2 had no impact on circCDYL2 expression as well. In addition, anti-HER2 antibody could not enrich the circCDYL2 (Fig. S3A). All data indicates no direct regulation between circCDYL2 and HER2 gene.

Then we detected whether circCDYL2 could interact with protein in HER2-associated pathways. Firstly, we successfully established circRNA pull-down assay using biotinylated probes specifically targeting circCDYL2 junction sequence (Fig. S3B). CircCDYL2-binding proteins were enriched by circRNA pull-down, and then performed by silver staining (Fig. 4E). Three proteins were identified by twice Mass spectrum (MS) (Table S6). GRB7 protein was enriched after circRNA pull-down using a circCDYL2 biotinylated probe (Fig. 4F). Furthermore, RIP assays using anti-GRB7 antibody could also enrich circCDYL2, indicating that circCDYL2 could interact with GRB7 protein (Fig. 4G). To find out the potential binding sites, we tested the interaction between circCDYL2 and recombinant GRB7 protein by RNA pull-down assay. The full-length circCDYL2 RNA product was truncated to five segments (sequence shown in Table S7). We found that segments 3, 4 and 5 but not segments 1 and 2 could pull down recombinant GRB7 protein, suggesting that the 1 nt ~ 360 nt of circCDYL2 was the binding region with GRB7 (Fig. S3C). In the presence of trastuzumab, silencing GRB7 using siRNA could reduce the phosphorylation of AKT and ERK1/2 under over-express circCDYL2, but had no effect on total protein level of AKT and ERK1/2 (Fig. 4H). CCK8 assay showed that trastuzumab-sensitive effect after GRB7 silencing could be partially restored by overexpression of circCDYL2 in both P-BT474 and P-SK-BR-3 cells (Fig. 4I), indicating that circCDYL2 promoted drug resistance of HER2^+^ BC cells via GRB7.

### circCDYL2 inhibits proteolytic ubiquitination of GRB7

**Fig. 5:**
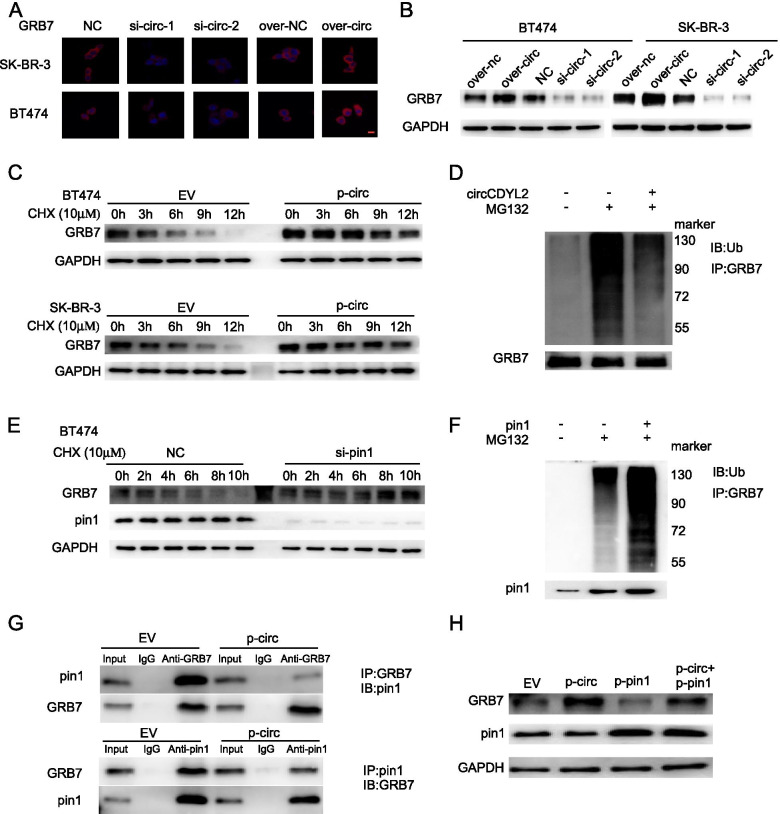
circCDYL2 modulated GRB7 protein stability by Pin1. **A**, **B** GRB7 protein expression in BT474 and SK-BR-3 cells after silencing and overexpression circCDYL2 by Immunofluorescence (**A**) and Western Blot (**B**). **C** Western Blot of GRB7 protein in BT474 and SK-BR-3 cells after circCDYL2 over-expressing by over-expressing lentivirus treated with CHX (10 μM) at indicated time points. EV: empty vector; p-circ: over-expressing circCDYL2. **D** SK-BR-3 cells after circCDYL2 overexpression were subject to IP with anti-GRB7 antibody followed by Western Blot. **E** Western Blot of GRB7 protein in BT474 and SK-BR-3 cells after knock downing pin1 treated with CHX (10 μM) at indicated time points. **F** SK-BR-3 cells after knock downing pin1 were subject to IP with anti-GRB7 antibody followed by Western Blot. **G** SK-BR-3 cells after circCDYL2 overexpression were subject to IP with anti-GRB7 antibody or anti-pin1 followed by Western Blot. **H** GRB7 protein expression in SK-BR-3 cells after overexpression pin1 with or without overexpressing circCDYL2 by western blot

Interestingly, we also found that the protein level of GRB7 was increased after circCDYL2 overexpression and decreased after silencing circCDYL2 in BT474 and SK-BR-3 cells (Fig. 5A, B). However, knocking down circCDYL2 in BT474 and SK-BR-3 cells did not result in significant change of GRB7 mRNA expression (Fig. S3D). Also, silencing GRB7 did not affect the RNA expression level of circCDYL2 (Fig. S3E). These results suggested that the interaction of circCDYL2 with GRB7 might contribute to post-transcriptional upregulation of GRB7. As shown in Fig. 5C, CHX (Cycloheximide, a protein synthesis inhibitor) treatment resulted in more stable of GRB7 in circCDYL2-overexpressing SK-BR-3 and BT474 cells. Furthermore, we sought to explore the underlying mechanism by which circCDYL2 stabilized GRB7 protein. As GRB7 was reported to be degraded by proteolytic ubiquitination [29], we hypothesised that circCDYL2 might interact with GRB7, which influenced the ubiquitination level of GRB7. The level of GRB7 ubiquitination was significantly decreased after overexpressing circCDYL2 in SK-BR-3 cells (Fig. 5D). Furthermore, we found that the expression of GRB7 protein displayed a positive correlation with circCDYL2 in HER2+ breast cancer patient tissues who treated with anti-HER2 therapy (Fig. S3F, R = 0.65, *P* < 0.001). In addition, Kaplan-Meier survival analysis in 127 HER2+ BC patients showed that HER2+ patients with high GRB7 had shorter DFS compared to those with low GRB7 (Fig. S3G, HR = 3.275, 95%CI = 1.724–6.22, *P* < 0.001). As reported, GRB7 protein stability modulated by Pin1 [29]. We found that GRB7 protein stability increased with the knockdown of Pin1 (Fig. 5E). Furthermore, we found that co-transfection of Pin1 with GRB7 increased ubiquitinated GRB7 protein (Fig. 5F). Western Blotting analysis indicated that anti-GRB7 could immuno-precipitated more abundant Pin1 protein in cells over-expressing circCDYL2. Meanwhile, anti-Pin1 antibody pulled down more GRB7 protein in circCDYL2-overexpressing cells (Fig. 5G). Over-expressing circCDYL2 could increase the expression of GRB7 protein under overexpressing Pin1 (Fig. 5H). Together, these results above suggest that circCDYL2 stabilizes GRB7 by inhibiting GRB7 ubiquitination, resulting in up-regulation of GRB7 protein in HER2+ breast cancer cells.

### circCDYL2 sustains activation of HER2 signaling by inducing GRB7/FAK interaction

**Fig. 6:**
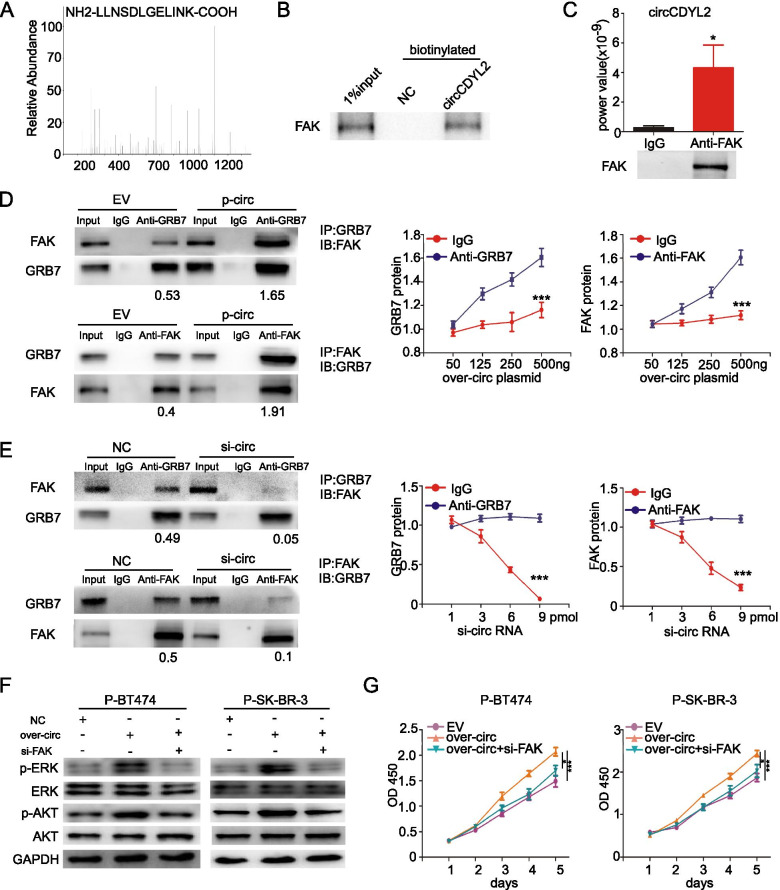
circCDYL2 enhances the formation of circCDYL2-GRB7-FAK complex. **A** Mass spectrogram of FAK protein. **B** Biotinylated circCDYL2 probe pull down results for FAK protein by Western Blot. **C** qRT-PCR analysis of circCDYL2 in RNA sample after RIP assay by anti-FAK antibody. **D** SK-BR-3 cells after circCDYL2 overexpression were subject to IP with anti-GRB7 antibody or anti-FAK followed by Western Blot. **E** SK-BR-3 cells after circCDYL2 silencing were subject to IP with anti-GRB7 antibody or anti-FAK followed by Western Blot. **F** Total and phosphorylased of AKT and ERK1/2 expression in both P-BT474 and P-SK-BR-3 cells, as detected by Western Blot after co-transfection with circCDYL2-overexpressing plasmid or FAK siRNA with Trastuzumab. **G** The proliferation of P-BT474 and P-SK-BR-3 cells after co-transfection circCDYL2-overexpressing plasmid or FAK siRNA with Trastuzumab, as detected by CCK8 assay

GRB7 is an adaptor protein that facilitates HER2-mediated downstream signal transduction through activating the phosphorylation of AKT and ERK [30]. As reported, the binding of FAK to GRB7 sustains activation of AKT and ERK signaling [31]. According to MS, circCDYL2 probe could pull down FAK protein (Fig. 6A). FAK protein was enriched after circRNA pull-down using a circCDYL2 biotinylated probe (Fig. 6B). Furthermore, RIP assays using anti-FAK antibody could also enrich circCDYL2, indicating that circCDYL2 could interact with FAK protein (Fig. 6C). To analyze whether circCDYL2 promoted binding GRB7 of FAK, western blotting analysis indicated that anti-GRB7 could immuno-precipitated more abundant FAK protein in cells over-expressing circCDYL2. Meanwhile, anti-FAK antibody pulled down more GRB7 protein in circCDYL2-overexpressing cells (Fig. 6D). Knock-down circCDYL2 could reduce the binding of FAK and GRB7 protein (Fig. 6E). Similar results were also found in JIMT-1 cells (Fig. S3H). FISH and IF assays further confirmed that GRB7 and circCDYL2 were mainly co-localized in the cytoplasm circCDYL2, and part of GRB7 and FAK protein were co-localized in the membrane of SK-BR-3 cells (Fig. S3I). In the presence of trastuzumab, silencing FAK using siRNA also could decrease the phosphorylation of AKT and ERK1/2 under over-expressing circCDYL2, but had no effect on total protein level of AKT and ERK1/2 (Fig. 6F). CCK8 assay showed that trastuzumab-sensitive effect after FAK silencing could be partially restored by over-expressing circCDYL2 in both P-BT474 and P-SK-BR-3 cells (Fig. 6G), indicating that circCDYL2 promoted drug resistance of HER2^+^ BC cells via FAK. These results suggest that circCDYL2, GRB7 and FAK protein can form a complex.

### Inhibition of FAK or GRB7 reduces circCDYL2-induced trastuzumab resistance

**Fig. 7:**
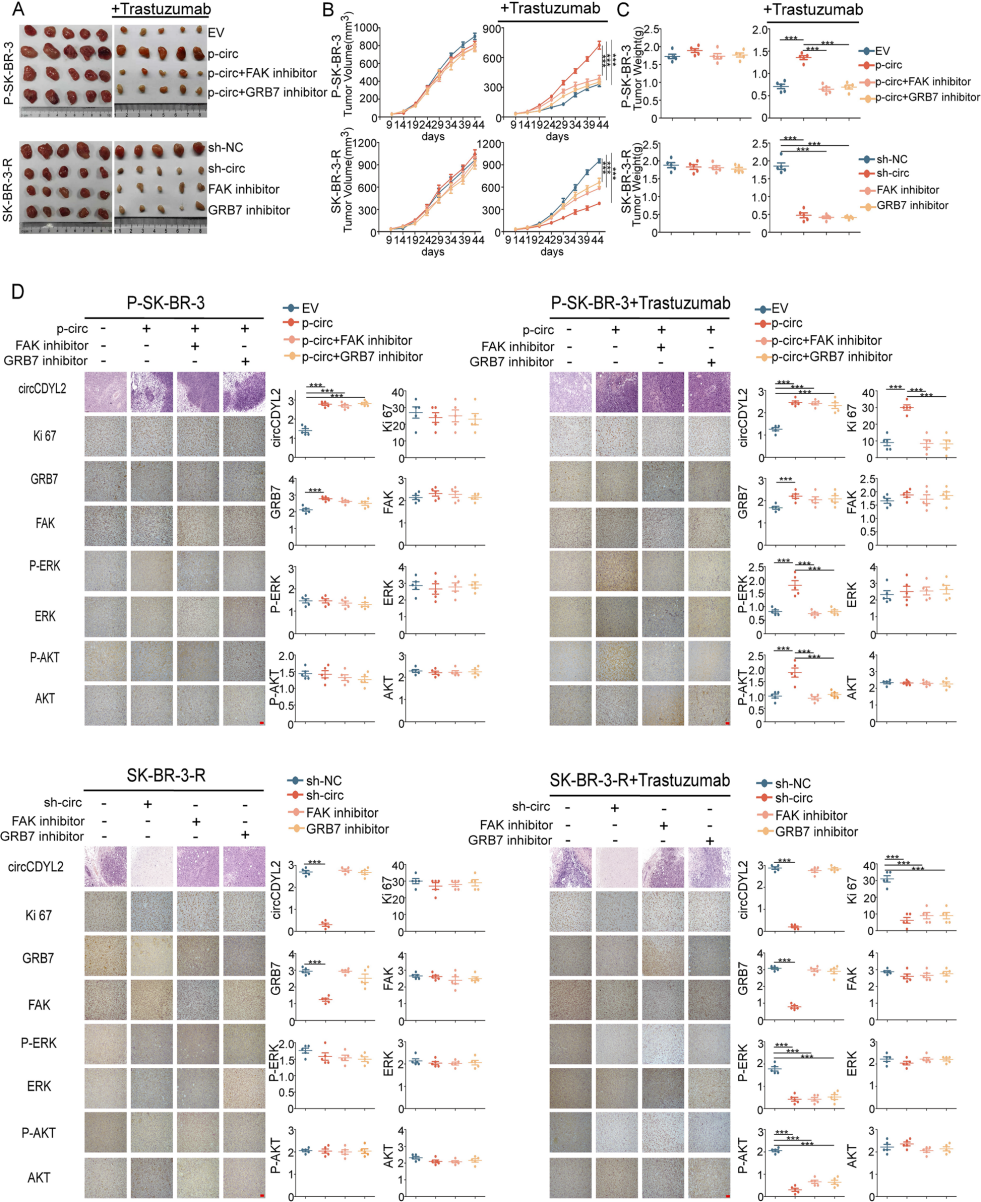
circCDYL2 promotes trastuzumab resistant of BC cells. **A** Representative images of tumors, which were formed by empty vector- or circCDYL2-transduced P-SK-BR-3 (upper) and negative control- or circCDYL2-knockdown SK-BR3-R (lower) cell lines, treated with either FAK inhibitor or GRB7 inhibitor combined with or without trastuzumab. **B** The volume of indicated tumors was measured on the indicated days. **C** The weight of indicated tumors were measured after tumor excision. **D** IHC staining of Ki-67, GRB7, FAK, P-ERK, ERK, P-AKT and AKT and ISH detection of circCDYL2 in the indicated tumors. Scale bar, 50 μm. *n* = 5 animals per group

To further investigate whether FAK and GRB7 contributed to circCDYL2-induced trastuzumab resistance of HER2^+^-BC, we designed relative in vivo experiments. Firstly, we stably over-expressed circCDYL2 using over-express lentivirus in P-SK-BR-3 cells and knocked down circCDYL2 using shRNA lentivirus. As shown in Fig. S3J, neither over-expressing nor knock-down circCDYL2 has effect on linear CDYL2 expression in P-SK-BR-3cells or SK-BR-3-R cells. P-SK-BR-3cells or SK-BR-3-R cells (1 × 10^7^ cells) were injected directly into the mammary fat pad of mice orthotopically (*n* = 5/group), accompanying with or without trastuzumab (10 mg/kg) treatment twice a week. The tumour volume was dynamically measured during forty-four days after injection. Without trastuzumab treating, over-express circCDYL2 in P-SK-BR-3 cells barely promoted tumor proliferation as compared with negative control, and knock-down circCDYL2 minimally inhibited tumor growth in SK-BR-3-R cells. Although trastuzumab did not influence the growth rate of the resistant SK-BR-3 xenografts, following know-down circCDYL2, the magnitude of tumor inhibition was significantly more profound in animals receiving trastuzumab dosing than those without treatment with the antibody, suggesting that circCDYL2 inhibition may sensitize trastuzumab-resistant breast cancer to the antibody. On the other hand, treatment with trastuzumab reduced the growth of tumor in nude mice inoculated with parental SK-BR-3 cells, and overexpressing circCDYL2 could rescue the tumor inhibition by trastuzumab treatment. More interesting, treated with FAK or GRB7 inhibitor could promote the effect of tumor inhibition by knock-downing circCDYL2. Meanwhile, FAK and GRB7 inhibitor also could rescue the trastuzumab resistant by over-expressing circCDYL2 (Fig. 7A-C). ISH and IHC were carried out to determine the level of circCDYL2, GRB7, FAK and Ki67 expression in tumors, and we also performed the activation of PI3K/AKT and MERK/ERK pathways (Fig. 7D). All these results indicate that circCDYL2 contributes to trastuzumab resistance of HER2^+^-breast cancer, either FAK inhibitor or GRB7 inhibitor reduces circCDYL2-induced trastuzumab resistance through the activation of PI3K/AKT and MERK/ERK pathways.

## Discussion

Nowadays chemotherapy combined with anti-HER2 monoclonal antibody such as trastuzumab greatly improves the survival of HER2^+^ breast cancer patients [32]. However, 25–30% of HER2^+^ patients still suffer recurrence after standard therapy [33], and the mechanisms underlying trastuzumab resistance remain largely unclear. Recently, multiple studies have demonstrated that circRNAs play crucial roles in drug resistance in malignant tumors including breast cancer. For instance, it was reported that upregulated hsa_circ_0006528 contributed adriamycin-resistant in breast cancer via the miR-1236-3p/CHD4 axis and circABCB10 was overexpressed in breast cancer that was involved in paclitaxel resistance through the circRNA/let-7a-5p/DUSP7 axis [34, 35]. Moreover, Sang et al. demonstrated that hsa_circ_0025202 functioned as a tumor suppressor circRNA in HR-positive breast cancer, which increased sensitivity of breast cancer to tamoxifen treatment via regulating the miR-182-5p/FOXO3a axis [36]. Importantly, li and colleagues identified an oncogenic circRNA, circRNA hsa_circ_0001598, which played a vital role in regulation of PD-L1 expression, resulted in immune escape and resistance of trastuzumab in breast cancer [37]. Herein, we found that HER2+ BC patients with high levels of circCDYL2 developed rapid recurrence and had shorter disease-free survival (DFS) and overall survival (OS) following anti-HER2 therapy compared to those with low circCDYL2. Overexpression of circCDYL2 in confered trastuzumab resistance in breast cancer cells in vitro and in vivo. Mechanically, circCDYL2 stabilized GRB7 by preventing its ubiquitination degradation and enhanced its interaction with FAK, which thus sustained the activities of downstream AKT and ERK1/2. Importantly, Trastuzumab-resistance of HER2+ BC cells with high circCDYL2 could be reversed by FAK or GRB7 inhibitor. Therefore, our results provided further evidence that circRNAs played vital roles in progression and development of breast cancer.

Nowadays, few predictive markers were found for HER2^+^ BC patients with trastuzumab resistance, and our study was the first research to reveal that circCDYL2 and GRB7 were essential genes in regulating trastuzumab sensitivity of HER2^+^ breast cancer. Our clinical data indicated that HER2^+^ breast cancer patients with high expression levels of circCDYL2 had shorter DFS after standard trastuzumab treatment. In in vitro experiments, circCDYL2 was higher expressed in trastuzumab-resistant HER2^+^ BC cell than that in parent cell lines. In in vivo PDX animal experiments, the tumors derived from trastuzumab-resistant HER2^+^ BC patients exhibited higher expression of circCDYL2 than the trastuzumab-sensitive patients. From the data above, the expression of circCDYL2 exhibited potential in clinical decision on HER2^+^ BC patients. For example, HER2^+^ patients with higher circCDYL2 maybe need alternative targeted drugs such as T-DM1, and receive much closer follow-up. In addition, circCDYL2 was stable and abundant in HER2^+^ BC cells or tumor tissues, making circCDYL2 an ideal biomarker for evaluation of trastuzumab therapeutic efficacy.

Importantly, our study provided several novel strategies to reverse trastuzumab resistance. Firstly, in in vitro experiments, silencing expression of circCDYL2 in trastuzumab-resistant HER2^+^ BC cells led to the increasing susceptibility of trastuzumab. In in vitro PDX animal experiments, silencing circCDYL2 could reverse trastuzumab resistance of tumors derived from trastuzumab-resistant HER2^+^ BC patients. These results suggested that circCDYL2 was an important culprit of trastuzumab resistance, for which circCDYL2 could be a therapeutic target for trastuzumab-resistant HER2^+^ patients. Secondly, mechanism study indicated that FAK/GRB7-mediated signaling pathways, including PI3K/AKT or RAS/ERK signaling pathway, contributed to trastuzumab resistance of HER2^+^ BC [38–40]. In this study, circCDYL2 was an essential molecule in GRB7-FAK complex formation. Therefore, FAK or GRB7 inhibitors were feasible to increase sensitivity of trastuzumab or even reverse trastuzumab resistance of the HER2^+^ BC patients with high expression of circCDYL2. Thirdly, tumors with trastuzumab resistance existed activation of both PI3K/AKT and RAS/ERK signaling pathway, therefore, drug or molecule targeted single pathway was not efficient to reverse trastuzumab resistance.

Several interesting discoveries in mechanism of circCDYL2 were found in this study. Firstly, we found that silencing circCDYL2 expression led to the up-regulation of GRB7 protein. As detected by qRT-PCR, the RNA level of GRB7 did not change after silencing circCDYL2, suggesting circCDYL2 did not regulate the transcription of GRB7 genes. Given that circCDYL2 directly binded to GRB7, circCDYL2 may inhibit the degradation of GRB7. Indeed, mechanical study indicated that GRB7 interacts with circCDYL2, thus to escape from digestion by ubiquitin-lysosomal pathway. It had been reported that GRB7 protein stability was regulated by the peptidyl-prolylcis/transisomerase (Pin1), and Pin1 increases GRB7 ubiquitination and degradation through proteasome-dependent proteolysis [29]. Numerous researches indicated that Pin1 was a tumor-promoting gene in breast cancer by promoting proliferation, migration, metabolism of cancer cells. Pin1 was highly expressed in HER2^+^ breast cancer, but out of expect, GRB7 was still stable without over-degradation. By co-IP experiments, the binding of Pin1 and GRB7 was impaired after over-expression of circCDYL2 in HER2^+^ BC cells, indicating that circCDYL2 played a role in separating the combination of Pin1 and GRB7 protein. In addition, how GRB7 binded to FAK was unclear before this research. CircRNA pull down assay and RIP assays indicated circCDYL2 directly binded to both GRB7 and FAK protein, and together formed circCDYL2-GRB7-FAK complex. When over-expression of circCDYL2 in BC cells, the binding of GRB7 and FAK was strengthened, suggesting that circCDYL2 acted as scaffold and promoted the binding of GRB7 and FAK.

## Conclusion

In summary, we identified a circRNA circCDYL2 that played a critical role in trastuzumab resistance in HER2^+^ BC patients. Mechanistically, we demonstrated that circCDYL2-GRB7-FAK complex played a critical role in HER2^+^ breast cancer cells and contributed to the progression of HER2^+^ breast cancer. Clinically, circCDYL2 could be used as a new molecule for predicting the prognosis of HER2^+^ BC patients, and we proved that FAK or GRB7 inhibitor could be used to reverse trastuzumab resistance of HER2^+^ BC patients (Fig. S4).

## Supplementary Information

**Additional file 1.**

## Data Availability

The datasets obtained and analyzed during the current study were made available from the corresponding authors through request.

## Abbreviations

HER2: Human Epidermal Growth Factor Receptor-2
circRNA: circular RNA
BC: Breast cancer
GRB7: Growth Factor Receptor Bound Protein 7
FAK: Focal adhesion kinase 1
SYSMH: Sun Yat-sen Memorial Hospital
IHC: Immunohistochemical staining
IF: Immunofluorescence
qRT-PCR: Real-Time Quantitative Reverse Transcription PCR
NC: Negative control
HR: Hazard ratio
95% CI: 95% confidence interval

## References

[1] Sung H, Ferlay J, Siegel RL, Laversanne M, Soerjomataram I, Jemal A, et al. Global cancer statistics 2020: GLOBOCAN estimates of incidence and mortality worldwide for 36 cancers in 185 countries. CA Cancer J Clin. 2021;71:209–49. 10.3322/caac.21660 33538338

[2] Slamon DJ, Clark GM, Wong SG, Levin WJ, Ullrich A, McGuire WL (1987). Human breast cancer: correlation of relapse and survival with amplification of the HER-2/neu oncogene. Science.

[3] Romond EH, Perez EA, Bryant J, Suman VJ, Geyer CJ, Davidson NE, Tan-Chiu E, Martino S, Paik S, Kaufman PA (2005). Trastuzumab plus adjuvant chemotherapy for operable HER2-positive breast cancer. N Engl J Med.

[4] Vogel CL, Cobleigh MA, Tripathy D, Gutheil JC, Harris LN, Fehrenbacher L, Slamon DJ, Murphy M, Novotny WF, Burchmore M (2002). Efficacy and safety of trastuzumab as a single agent in first-line treatment of HER2-overexpressing metastatic breast cancer. J Clin Oncol.

[5] Harbeck N, Huang C, Hurvitz S, Yeh D, Shao Z, Im S, Jung KH, Shen K, Ro J, Jassem J (2016). Afatinib plus vinorelbine versus trastuzumab plus vinorelbine in patients with HER2-overexpressing metastatic breast cancer who had progressed on one previous trastuzumab treatment (LUX-breast 1): an open-label, randomised, phase 3 trial. Lancet Oncol.

[6] Dawood S, Broglio K, Buzdar AU, Hortobagyi GN, Giordano SH (2010). Prognosis of women with metastatic breast Cancer by HER2 status and Trastuzumab treatment: An institutional-based review. J Clin Oncol.

[7] Cameron D, Piccart-Gebhart MJ, Gelber RD, Procter M, Goldhirsch A, de Azambuja E, Castro G, Untch M, Smith I, Gianni L (2017). 11 years' follow-up of trastuzumab after adjuvant chemotherapy in HER2-positive early breast cancer: final analysis of the HERceptin adjuvant (HERA) trial. Lancet.

[8] Dave B, Migliaccio I, Gutierrez MC, Wu MF, Chamness GC, Wong H, Narasanna A, Chakrabarty A, Hilsenbeck SG, Huang J (2011). Loss of phosphatase and tensin homolog or phosphoinositol-3 kinase activation and response to trastuzumab or lapatinib in human epidermal growth factor receptor 2-overexpressing locally advanced breast cancers. J Clin Oncol.

[9] Saini KS, Loi S, de Azambuja E, Metzger-Filho O, Saini ML, Ignatiadis M, Dancey JE, Piccart-Gebhart MJ (2013). Targeting the PI3K/AKT/mTOR and Raf/MEK/ERK pathways in the treatment of breast cancer. Cancer Treat Rev.

[10] Serra V, Scaltriti M, Prudkin L, Eichhorn PJA, Ibrahim YH, Chandarlapaty S, Markman B, Rodriguez O, Guzman M, Rodriguez S (2011). PI3K inhibition results in enhanced HER signaling and acquired ERK dependency in HER2-overexpressing breast cancer. Oncogene.

[11] Rexer BN, Ghosh R, Narasanna A, Estrada MV, Chakrabarty A, Song Y, Engelman JA, Arteaga CL (2013). Human breast Cancer cells harboring a gatekeeper T798M mutation in HER2 overexpress EGFR ligands and are sensitive to dual inhibition of EGFR and HER2. Clin Cancer Res.

[12] Li J, Xiao Q, Bao Y, Wang W, Goh J, Wang P, Yu Q (2019). HER2-L755S mutation induces hyperactive MAPK and PI3K-mTOR signaling, leading to resistance to HER2 tyrosine kinase inhibitor treatment. Cell Cycle.

[13] Kataoka Y, Mukohara T, Shimada H, Saijo N, Hirai M, Minami H (2010). Association between gain-of-function mutations in PIK3CA and resistance to HER2-targeted agents in HER2-amplified breast cancer cell lines. Ann Oncol.

[14] Sahin O, Wang Q, Brady SW, Ellis K, Wang H, Chang CC, Zhang Q, Priya P, Zhu R, Wong ST (2014). Biomarker-guided sequential targeted therapies to overcome therapy resistance in rapidly evolving highly aggressive mammary tumors. Cell Res.

[15] Zhong Y, Du Y, Yang X, Mo Y, Fan C, Xiong F, et al. Circular RNAs function as ceRNAs to regulate and control human cancer progression. Mol Cancer. 2018;17:79–89. 10.1186/s12943-018-0827-8 PMC5889847 29626935

[16] Jeck WR, Sharpless NE (2014). Detecting and characterizing circular RNAs. Nat Biotechnol.

[17] Memczak S, Jens M, Elefsinioti A, Torti F, Krueger J, Rybak A, Maier L, Mackowiak SD, Gregersen LH, Munschauer M (2013). Circular RNAs are a large class of animal RNAs with regulatory potency. Nature.

[18] Vo JN, Cieslik M, Zhang Y, Shukla S, Xiao L, Zhang Y, Wu Y, Dhanasekaran SM, Engelke CG, Cao X (2019). The landscape of circular RNA in Cancer. Cell.

[19] Liu Z, Zhou Y, Liang G, Ling Y, Tan W, Tan L, et al. Circular RNA hsa_circ_001783 regulates breast cancer progression via sponging miR-200c-3p. Cell Death Dis. 2019;10:55–68. 10.1038/s41419-018-1287-1 PMC6343010 30670688

[20] Liang G, Ling Y, Mehrpour M, Saw PE, Liu Z, Tan W, et al. Autophagy-associated circRNA circCDYL augments autophagy and promotes breast cancer progression. Mol Cancer. 2020;19:65–80. 10.1186/s12943-020-01152-2 PMC7093993 32213200

[21] Yang Y, Gao X, Zhang M, Yan S, Sun C, Xiao F, Huang N, Yang X, Zhao K, Zhou H (2018). Novel role of FBXW7 circular RNA in repressing Glioma tumorigenesis. J Natl Cancer Inst.

[22] Huang A, Zheng H, Wu Z, Chen M, Huang Y (2020). Circular RNA-protein interactions: functions, mechanisms, and identification. Theranostics.

[23] Zhu Y, Zou C, Jia Y, Zhang H, Ma X, Zhang J (2020). Knockdown of circular RNA circMAT2B reduces oxygen-glucose deprivation-induced inflammatory injury in H9c2 cells through up-regulating miR-133. Cell Cycle (Georgetown, Tex.).

[24] Sang L, Ju H, Liu G, Tian T, Ma G, Lu Y, Liu Z, Pan R, Li R, Piao H (2018). LncRNA CamK-A regulates Ca2+−signaling-mediated tumor microenvironment remodeling. Mol Cell.

[25] Zhang X, Wang S, Wang H, Cao J, Huang X, Chen Z, et al. Circular RNA circNRIP1 acts as a microRNA-149-5p sponge to promote gastric cancer progression via the AKT1/mTOR pathway. Mol Cancer. 2019;18:20–43. 10.1186/s12943-018-0935-5 PMC6360801 30717751

[26] Zhou L, Zhai M, Huang Y, Xu S, An T, Wang Y, Zhang R, Liu C, Dong Y, Wang M (2019). The circular RNA ACR attenuates myocardial ischemia/reperfusion injury by suppressing autophagy via modulation of the Pink1/ FAM65B pathway. Cell Death Differ.

[27] Yang C, Yuan W, Yang X, Li P, Wang J, Han J, et al. Circular RNA circ-ITCH inhibits bladder cancer progression by sponging miR-17/miR-224 and regulating p21, PTEN expression. Mol Cancer. 2018;17:19–30. 10.1186/s12943-018-0771-7 PMC5793418 29386015

[28] Du WW, Yang W, Liu E, Yang Z, Dhaliwal P, Yang BB (2016). Foxo3 circular RNA retards cell cycle progression via forming ternary complexes with p21 and CDK2. Nucleic Acids Res.

[29] Tai Y, Tung L, Lin Y, Lu P, Chu P, Wang M, Huang W, Chen K, Lee H, Shen T (2016). Grb7 protein stability modulated by Pin1 in association with cell cycle progression. PLoS One.

[30] Nencioni A, Cea M, Garuti A, Passalacqua M, Raffaghello L, Soncini D, Moran E, Zoppoli G, Pistoia V, Patrone F, Ballestrero A (2010). Grb7 upregulation is a molecular adaptation to HER2 signaling inhibition due to removal of Akt-mediated gene repression. PLoS One.

[31] Chu P, Tai Y, Shen T (2019). Grb7, a critical mediator of EGFR/ErbB signaling, in Cancer development and as a potential therapeutic target. Cells-Basel.

[32] Gianni L, Pienkowski T, Im YH, Tseng LM, Liu MC, Lluch A, Staroslawska E, de la Haba-Rodriguez J, Im SA, Pedrini JL (2016). 5-year analysis of neoadjuvant pertuzumab and trastuzumab in patients with locally advanced, inflammatory, or early-stage HER2-positive breast cancer (NeoSphere): a multicentre, open-label, phase 2 randomised trial. Lancet Oncol.

[33] Piccart M, Procter M, Fumagalli D, de Azambuja E, Clark E, Ewer MS, et al. Adjuvant Pertuzumab and Trastuzumab in early HER2-positive breast Cancer in the APHINITY trial: 6 Years' follow-up. J Clin Oncol. 2021;39:1448–57. 10.1200/JCO.20.01204 33539215

[34] Hao J, Du X, Lv F, Shi Q (2021). Knockdown of circ_0006528 suppresses cell proliferation, migration, invasion, and Adriamycin Chemoresistance via regulating the miR-1236-3p/CHD4 Axis in breast Cancer. J Surg Res.

[35] Yang W, Gong P, Yang Y, Yang C, Yang B, Ren L (2020). Circ-ABCB10 contributes to paclitaxel resistance in breast Cancer through let-7a-5p/DUSP7. Axis..

[36] Sang Y, Chen B, Song X, Li Y, Liang Y, Han D, Zhang N, Zhang H, Liu Y, Chen T (2019). circRNA_0025202 regulates Tamoxifen sensitivity and tumor progression via regulating the miR-182-5p/FOXO3a Axis in breast Cancer. Mol Ther.

[37] Huang L, Ma J, Cui M. Circular RNA hsa_circ_0001598 promotes programmed death-ligand-1-mediated immune escape and trastuzumab resistance via sponging miR-1184 in breast cancer cells. Immunol Res. 2021;69:558–67. 10.1007/s12026-021-09237-w 34559381

[38] Sang J, Kulkarni K, Watson GM, Ma X, Craik DJ, Henriques ST, Poth AG, Benfield AH, Wilce JA (2019). Evaluation of cyclic peptide inhibitors of the Grb7 breast Cancer target: small change in cargo results in large change in cellular activity. Molecules.

[39] Shen TL, Guan JL (2001). Differential regulation of cell migration and cell cycle progression by FAK complexes with Src, PI3K, Grb7 and Grb2 in focal contacts. FEBS Lett.

[40] Chu P, Huang L, Hsu C, Liang C, Guan J, Hung T, Shen T (2009). Tyrosine phosphorylation of growth factor receptor-bound Protein-7 by focal adhesion kinase in the regulation of cell migration, proliferation, and tumorigenesis. J Biol Chem.

