# Supplementary material for: circCDYL2 promotes trastuzumab resistance via sustaining HER2 downstream signaling in breast cancer
Source: Mol Cancer. 2022 Jan 3;21:8. doi: 10.1186/s12943-021-01476-7 (PMC8722291; doi:10.1186/s12943-021-01476-7)
Supplement: Supplementary file 1 — Additional file 1. [file 12943_2021_1476_MOESM1_ESM.docx]

**Supplementary Data**

**Title:** circCDYL2 promotes trastuzumab resistance via sustaining HER2 downstream signaling in breast cancer

Yun Ling^1,5^*, Gehao Liang^1,6^*, Qun Lin^1^*, Xiaolin Fang^1^, Qing Luo^1^, Yinghuan Cen^1^, Maryam Mehrpour^3,4^, Ahmed Hamai^3,4^, Zihao Liu^1^, Yu Shi^1^, Juanmei Li^1^, Wanyi Lin^1^, Shijie Jia^1^, Wenqian Yang^1^, Qiang Liu^1^, Erwei Song^1^, Jun Li^1,2#^, Chang Gong^1#^

^1^Breast Tumor Center, Guangdong Provincial Key Laboratory of Malignant Tumor Epigenetics and Gene Regulation, Sun Yat-sen Memorial Hospital, Sun Yat-sen University, Guangzhou, 510120, China;

^2^Department of Biochemistry, Zhongshan School of Medicine, Sun Yat-sen University, Guangzhou, 510080, China;

^3^Institut Necker-Enfants Malades (INEM), Inserm U1151-CNRS UMR 8253, Paris, 75993, France;

^4^Université Paris Descartes-Sorbonne Paris Cité, Paris, 75993, France.

^5^Department of Breast Surgery, the Second Affiliated Hospital, Guangzhou Medical University, Guangzhou, 510120, China.

^6^Department of Breast Oncology, Sun Yat-sen University Cancer Center, Guangzhou, 510080, China.

**Correspondence to:**

Chang Gong, Breast Tumor Center, Sun Yat-sen Memorial Hospital, Sun Yat-sen University, 107 Yanjiang West Road, Guangzhou, 510120, P.R. China; Phone:+86-20-81333407, Fax:+86-20-81333407; Email: gchang@mail.sysu.edu.cn.

Jun Li, Department of Biochemistry, Zhongshan School of Medicine, Sun Yat-sen University, Guangzhou, 510080, P.R. China; Phone: +86(20)87335828. Fax: +86(20)87335828. E-mail: lijun37@mail.sysu.edu.cn.

* Authors contributed equally to this study.

**Inventory**

Supplementary Materials and Methods

1 Table

5 Supplementary Figures

10 Supplementary Tables

**Supplementary Materials and Methods**

**Cell culture and treatment**

Human breast cancer cell lines MDA-MB-231, MCF-7, MDA-MB-453, BT474, SK-BR-3 and JIMT-1 were obtained from American Type Culture Collection (ATCC). 3 pmol siRNA or 125 ng over-expressing plasmid (sequence shown in **Table S8**) was added to cells with 3.75 μl [lipofectamine 3000](https://www.sogou.com/link?url=DSOYnZeCC_owkDvmYG0gMz-JrNZwwuWK1VqBoQ46pYN0gohKX14THasYM9mIOXNn2uFhv1mjiPiuLaNMGv04nw..) (Invitrogen, MA, USA) and 250 μl Opti-MEM (Invitrogen, Carlsbad, USA). Total RNA or proteins were collected after transfection for 48 hours. Sh-circCDYL2 or lentivirus that overexpressing circCDYL2 (Hanbio, Shanghai, China) was used to establish SK-BR-3 cell lines stably knock-down or over-expressing circCDYL2.

Trastuzumab-resistant BT474-R, SK-BR-3-R and MDA-MB-453-R cells were established as reported elsewhere [1]. In our previous research [2], we treated HER2+ cell lines (P-SK-BR-3, P-MDA-MB-453, and P-BT474) with low-dose trastuzumab (5 μg/ml). After six months of cultivation, we obtain breast cancer cells with trastuzumab resistance (SK-BR-3-R, MDA-MB-453-R and BT474-R). JIMT-1 cells were insensitive to trastuzumab according previous research [3]. Trastuzumab was obtained from Sun Yat-sen Memorial Hospital (SYSMH). 2 μg/ml Trastuzumab was added to cells every day. 10 mg/kg Trastuzumab was injected into Balb/c nude twice a week.

**Cell Counting Kit-8 (CCK8)**

The CCK8 assay was performed by CCK8 assay kit manual (Dojindo, Japan). BT474 or SK-BR-3 or JIMT-1 cells were seeded in 96-well plates (5000 cells / well) . 10 μl CCK-8 solution was added to the cells and incubated for 2 hours at 37°C. The absorbance at 450 nm was measured by spectrophotometer.

**Colony formation assay**

BT474 or SK-BR-3 or JIMT-1 cells were seeded in 6-well plates (3000 cells / well) and cultured in a cell incubator for three weeks. The cells were fixed by 4% paraformaldehyde for 15 mins, and dyed by 1% crystal violet for 20 mins. The number of cell colonies was calculated by Image J (Wayne Rasband, National Institutes of Health, USA).

**Cell vitality assay**

BT474 and SK-BR-3 cells (5 $\times$ 10^4^ cells/ well) or JIMT cells (1 $\times$ 10^4^ cells/ well) with or without treatment were seeded in 12-well plates. For 5 continuous days, cells were digested and resuspended. 20 μl cell suspension was measured with the automatic cell counter to count the living cell numbers (Countstar Bio-tech, IC100, Shanghai, China).

**EdU assay**

The EdU assay was performed by EdU assay kit manual (RiboBio, China). BT474 or SK-BR-3 or JIMT-1 cells (5000 cells / well) were seeded in 96-well plates for one night. The next day, 25 μM EdU solution was added to the cells and incubated for 2 hours at 37°C. The cells were fixed by 4% paraformaldehyde for 15 mins, and stained by Apollo reaction solution for EdU and Hoechst 33342 for nucleus staining. The ratio of cell proliferation was calculated by Image J.

**RNA isolation and qRT-PCR**

The total RNA of breast cancer cells was extracted using TRIzol reagent (Invitrogen, Carlsbad, USA) and the total RNA of breast cancer tissues was extracted using Tissue RNA Purification Kit Plus (ES Science, China) according to the manufacturer's instruction. Quantitative Real-time reverse transcription-polymerase chain reaction (PCR) primers and probes were designed with the assistance of the Primer Express v 2.0 software (Applied BioSystems, Foster City, CA, USA). Expression data were normalized to the geometric mean of housekeeping gene β-actin to control the variability in expression levels and calculated as 2- ^[(Ct of gene) – (Ct of β-actin)]^, where Ct represents the cycle threshold for each transcript. The real-time quantitative polymerase chain reaction (qRT-PCR) was performed using SYBR qRT-PCR Master Mix (Vazyme Biotech, China) on Roche LightCycler 480 II system (Roche, Switzerland). All primers are listed in Table S10.

**Western Blot**

Cells were lysed in RIPA buffer (89900, Pierce, USA). The concentration of protein was detected by bicinchoninic acid (BCA) kit (30342, Cwbio, China). 20 μg protein was separated by 7.5%~10% SDS-PAGE and then transferred onto PVDF membrane (Millipore, Schwalbach, Germany). The PVDF membranes were blocked with 5% bovine serum albumin (BSA) at RT for 1 hour. Anti-GAPDH (8884, Cell Signaling Technology, 1:1000), anti-GRB7 (A5809, Abclonal, 1:1000), anti-pAKT (GTX32459, GeneTex, 1:1000), anti-AKT (4691, Cell Signaling Technology, 1:2000), anti-pERK (GTX24819, GeneTex, 1:1000), anti-ERK (4695, Cell Signaling Technology, 1:1000), anti-FAK antibody (3285, Cell Signaling Technology, USA, 1:1000) were incubated overnight at 4 ℃ respectively. A secondary antibody (Anti-Rabbit:7074S, Anti-Mouse:7076S, Cell Signaling Technology, 1:5000) was incubated for 1 hourat RT. Finally, the blots were detected by chemiluminescence kit (P90719, Millipore, USA) and analysed by Image Lab.

**circRNAs deep sequencing**

Total RNA was extracted from BC tissues with trastuzumab-resistant (n=5) and trastuzumab-sensitive (n=5) of HER2+ BC by Trizol (Invitrogen, USA). Epicenter Ribo-Zero rRNA Removal Kit (Illumina, CA, USA) and RNAse R (Epicenter, CA, USA) are applied to remove Ribosomal and linear RNA respectively. RNA-seq libraries were using the NEBNext® Ultra™ RNA Library Prep Kit (NEB, USA), and prepared to deep sequencing with an Illumina HiSeq X-ten at RiboBio Co. Ltd ( Guangzhou, China). When circRNA would be detected by CIRI2 and CIRCexplorer2, it will be scaled to RPM (Reads Per Million mapped reads) .

**Mass Spectrometry (MS)**

The gel stripes were separated and digested by gel electrophoresis and enzymatic. Sequentially, the peptides were extracted by acetonitrile, dissolved in 0.1% formic acid, and then were delivered onto a tandem self-packed C18 column. The liquid elution was prepared to mass spectrometry with a TripleTOF 5600 (SCIEX, USA) at BGI Co. Ltd (Shenzhen, China). Mascot (v2.3) software was used to analyze the raw file and search the Unipreview9606nr (20312 sequences) database to identified the proteins.

**Immunofluorescence (IF)**

The cells were seeded in a confocal dish and incubated with anti-GRB7 (A5809, Abclonal, 1:1000) or anti-FAK antibody (71433, Cell Signaling Technology, USA, 1:1000) overnight at 4℃. Next day, the cells were incubated with fluorescein secondary antibody (1:3000, FI-1000-1.5, Vector, Germany) for 1 hour at RT. Then cells were stained with DAPI for 10 mins at RT. Finally, the cells were observed by confocal microscope.

**Immunohistochemistry (IHC)**

IHC was performed on paraffin sections of breast cancer tissues or animal tumors using SP Rabbit & Mouse HRP Kit (Cwbio, China), according to manufacturer’s instruction. Anti-GRB7 (A5809, Abclonal, 1:100), anti-Ki67 (MBA-0672, MBX, 1:100), anti-pAKT (GTX32459, GeneTex, 1:100), anti-AKT (4691, Cell Signaling Technology, 1:100), anti-pERK (GTX24819, GeneTex, 1:100), anti-ERK (4695, Cell Signaling Technology, 1:100), and anti-FAK antibody (3285, Cell Signaling Technology, USA, 1:100) were used. The intensity and proportion at whole section were recorded on a scale of 0 (no staining), 1 (light yellow), 2 (yellow), and 3 (dark yellow). Total score of detected protein = ∑proportion * intensity score.

**References:**

1. Carr JR, Park HJ, Wang Z, Kiefer MM, Raychaudhuri P: **FoxM1 Mediates Resistance to Herceptin and Paclitaxel.** *Cancer Res* 2010, **70**:5054-5063.

2. Gong C, Yao Y, Wang Y, Liu B, Wu W, Chen J, Su F, Yao H, Song E: **Up-regulation of miR-21 Mediates Resistance to Trastuzumab Therapy for Breast Cancer**. *J Biol Chem* 2011, **286**:19127-19137.

3. Tanner M, Kapanen AI, Junttila T, Raheem O, Grenman S, Elo J, Elenius K, Isola J: **Characterization of a novel cell line established from a patient with Herceptin-resistant breast cancer**. *Mol Cancer Ther* 2004, **3**:1585-1592.

**
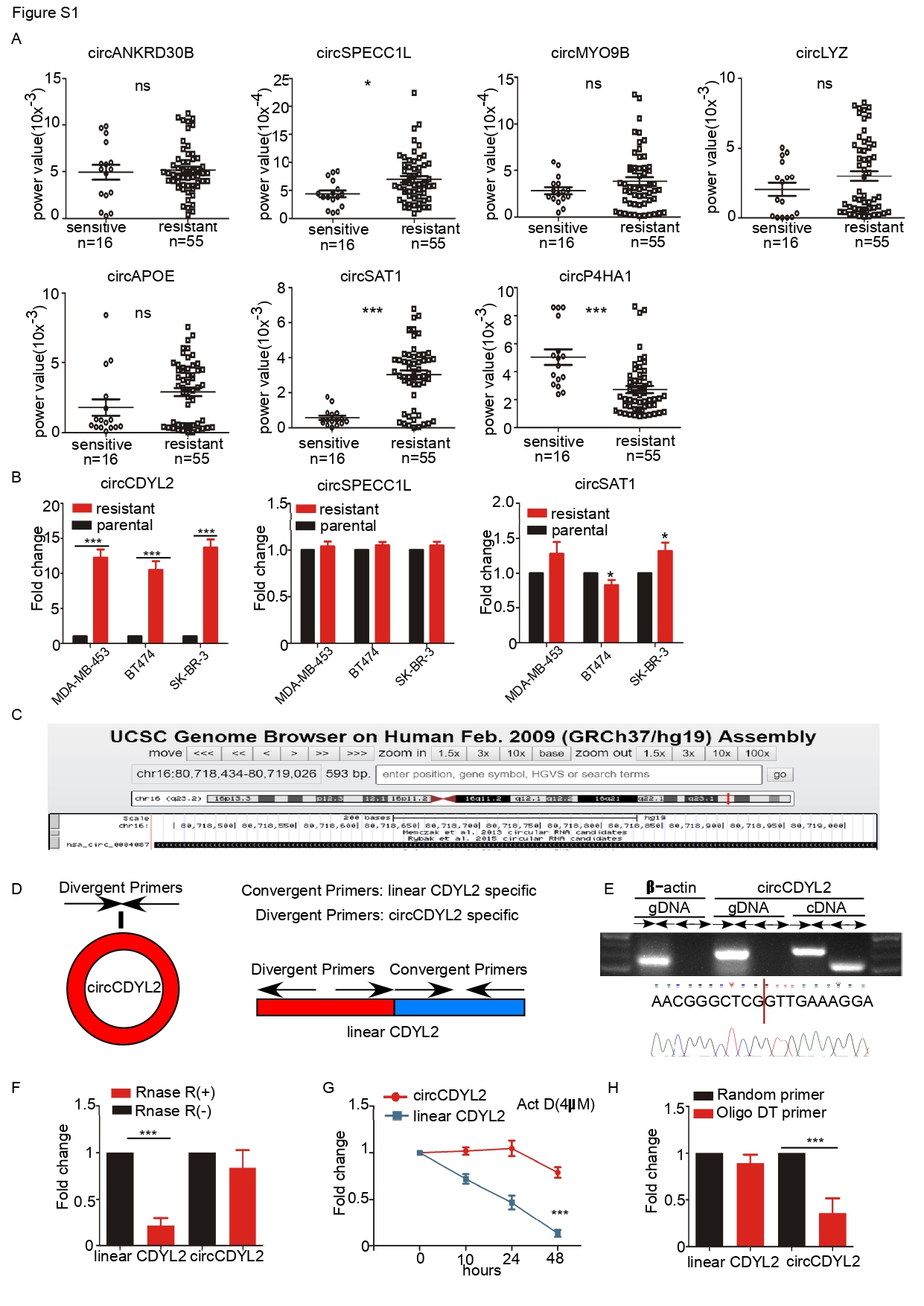
Supplementary Figure**

**Fig S1**. **Characterization of Trastuzumab-resistant associated circRNA expression profile in HER2^+^ BC patients. A.** Quantitative analysis of seven circRNAs expression in HER2^+^ cancer tissues with Trastuzumab sensitive (n=16) and Trastuzumab resistant (n=55) by qRT-PCR. **B.** qRT-PCR for the analysis of the existence of circRNAs in BC cells. **C.** Genomic loci of circCDYL2. **D.** Design of convergent and divergent primers. **E.** The genomic loci of CDYL2 gene and circCDYL2. Sanger sequencing of the back-splicing of circCDYL2. **F.** qRT-PCR analysis of circCDYL2 and linear CDYL2 with or without RNase R treatment. **G**. qRT-PCR quantification of linear CDYL2 and circCDYL2 in BT474 cells treated with Actinomycin D (4 µM) at indicated time points. **H**. qRT-PCR analysis of circCDYL2 and linear CDYL2 by using random primer or oligo DT primer. All experiments were repeated at least 3 times. *P< 0.05, **P< 0.01, ***P< 0.005. Error bars indicate S.E.M.

**
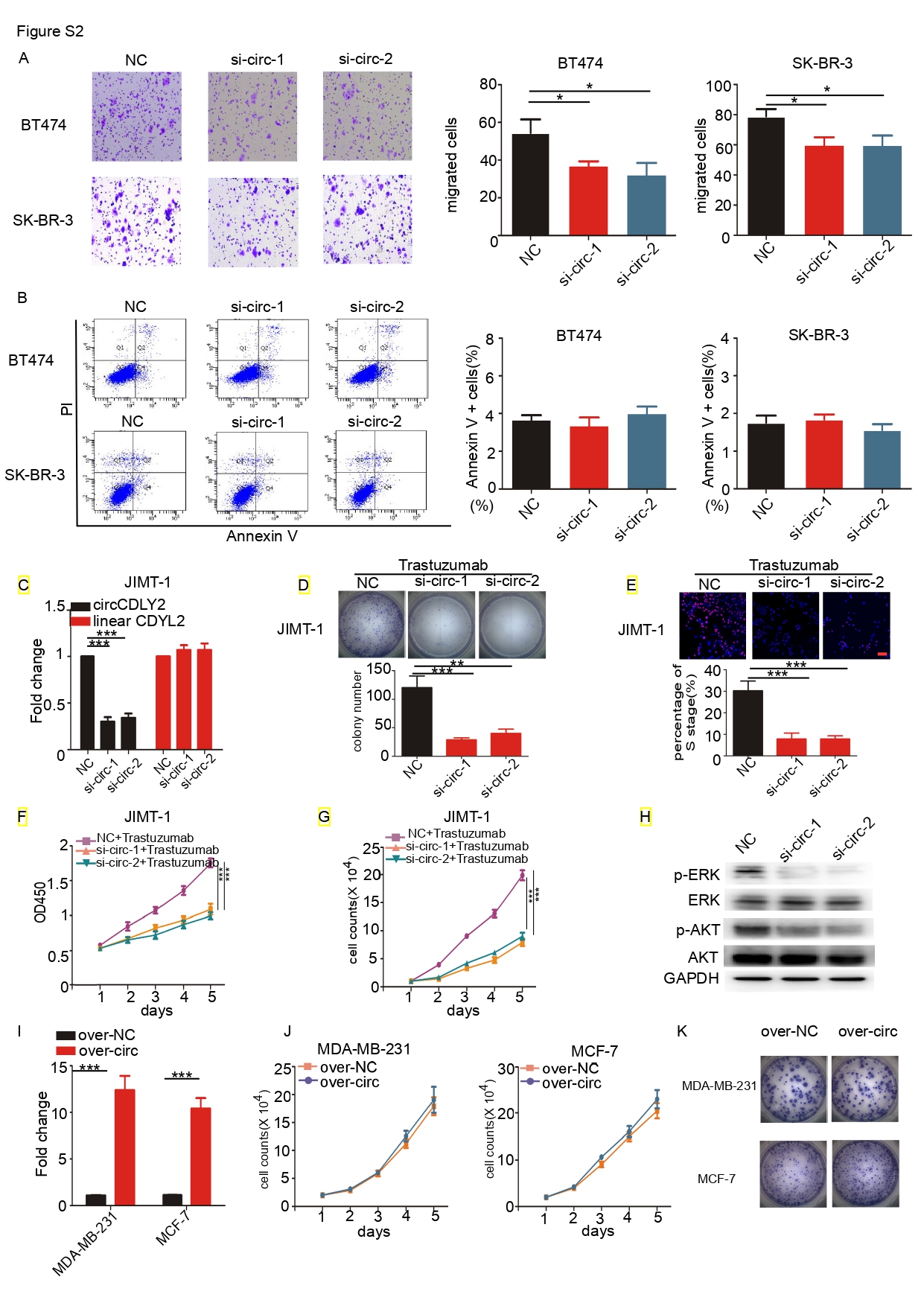
**

**Fig. S2**. **The functional role of circCDYL2 in HER2^+^ BC cells and HER2^-^ BC cells. A, B.** Migration assay (A) and PI/Annexin V double-staining analysis of apoptosis (B) in BT474 and SK-BR-3 cells after circCDYL2 silencing by circCDYL2 siRNA. **C**. qRT-PCR analysis of circCDYL2 and linear CDYL2 in JIMT-1 cells after circCDYL2 silencing or over-expressing by circCDYL2 specific siRNAand over-expressing plasmid. **D-G**. The cell viability of BC cells after treatments with circCDYL2 siRNA or over-expressing circCDYL2 plasmid or trastuzumab, as detected by colony formation (D), EdU assay (E), CCK8 assay (F), cell viability assay (G). **H**. Total and phosphorylated of AKT and ERK1/2 expression in JIMT-1 cells, as detected by Western Blot. **I.** qRT-PCR analysis of circCDYL2 in both MDA-MB-231 and MCF-7 after circCDYL2 over-expressing by circCDYL2 specific over-expressing plasmid. **J, K.** cell viability assay(J) and colony formation(K) in MDA-MB-231 and MCF-7 cells after transfecting plasmid overexpressing circCDYL2. All experiments were repeated at least 3 times. **P*< 0.05, ***P*< 0.01, ****P*< 0.005. Error bars indicate S.E.M.

**
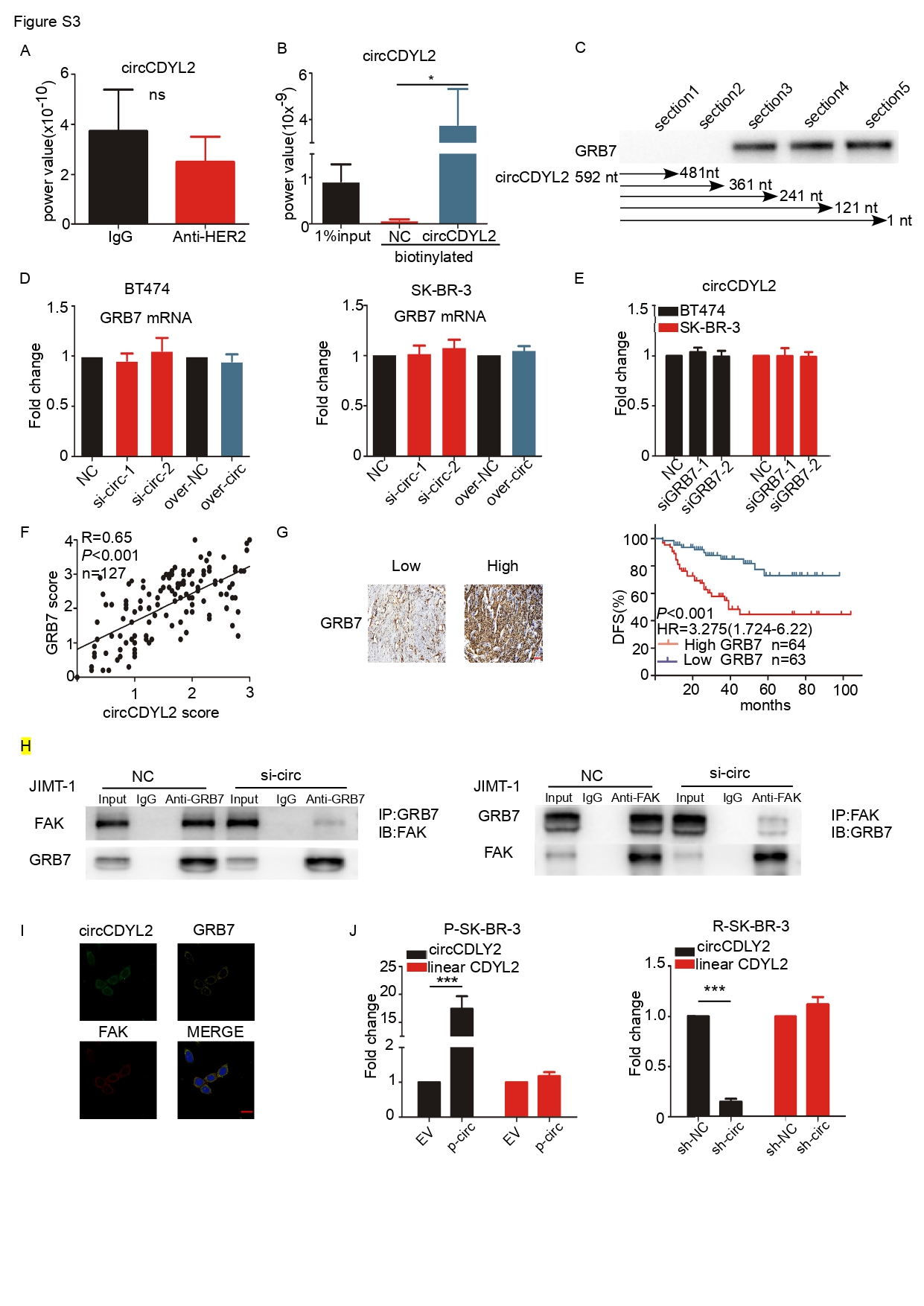
Fig. S3. The function role of GRB7 in HER2^+^ breast cancer cells. A.** qRT-PCR analysis of circCDYL2 in RNA sample after RIP assay by anti-HER2 antibody. **B**. circRNA pull-down assay with NC or circCDYL2 biotinylated probe and followed by qRT-PCR detection of circCDYL2. **C.** Qualitative analysis of GRB7 protein expression in pull down with different length products by Western Blot. **D.** GRB7 mRNA expression after silencing or overexpressing circCDYL2 in BT474 and SK-BR-3. **E.** qRT-PCR analysis of circCDYL2 in both BT474 and SK-BR-3 after GRB7 silencing. **F.** GRB7 protein expression was positively correlated with circCDYL2 in BC patient tissues. **G.** Analysis of the correlation between GRB7 expression and disease-free survival (DFS) and the representative images of GRB7 by IHC. Scale bar = 50 μm. **H.** JIMT-1 cells after circCDYL2 silencing were subject to IP with anti-GRB7 antibody or anti-FAK followed by Western Blot. **I.** Co-localization of circCDYL2, FAK and GRAB7 protein. Scale bar = 50 μm. **J.** RT-PCR analysis of circCDYL2 and linear CDYL2 in both P-SK-BR-3and SK-BR-3-R cells after circCDYL2 over-expressingor silencing by circCDYL2-overexpressing lentivirusand circCDYL2 shRNA lentivirus.

**
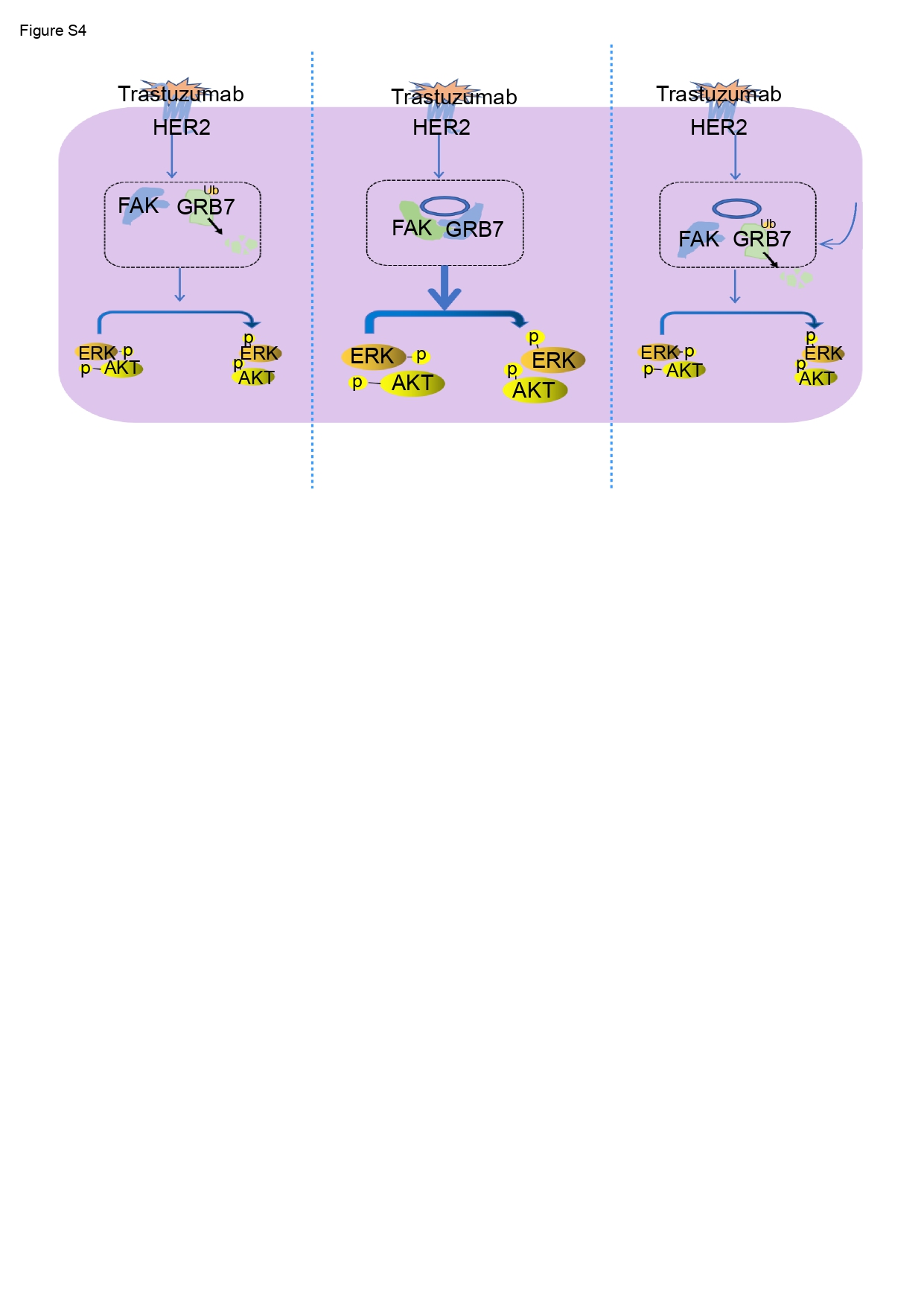
**

**Fig. S4. Graphical summary.** circCDYL2 acts as scaffold to promote the binding of GRB7 and GRB7, thus to sustain HER2 downstream signaling and lead to trastuzumab resistance of HER2^+^ BC patients. FAK or GRB7 inhibitor treatment may reverse trastuzumab resistance by inhibiting the activation of FAK or GRB7.

Supplement figure for reviwer only


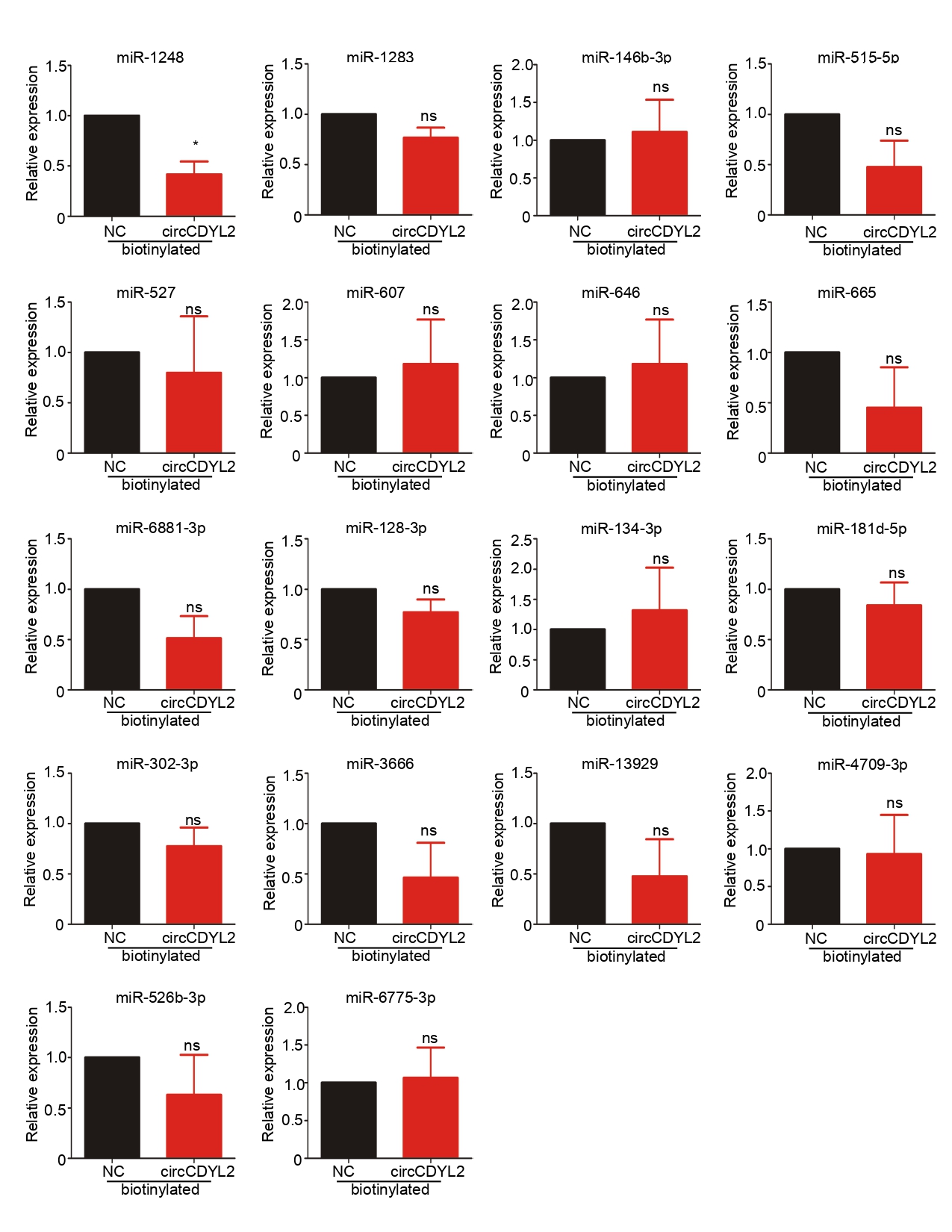


**Table 1. Patient characteristics stratified by circCDYL2 expression**

|  | **Number of patients** | | |  |  |
| --- | --- | --- | --- | --- | --- |
|  | **Low**  **circCDYL2** | **High**  **circCDYL2** | | **Total** | ***P* value** |
| **Age** |  | |  |  | 0.06 |
| >35 | 57 | | 50 | 107 |  |
| ≤35 | 6 | | 14 | 20 |  |
| **Menopause** |  | |  |  | 0.91 |
| Yes | 22 | | 23 | 45 |  |
| No | 41 | | 41 | 82 |  |
| **ER status** |  | |  |  | 0.06 |
| Positive (≥10%) | 39 | | 29 | 68 |  |
| Negative (<10%) | 24 | | 35 | 59 |  |
| **PR status** |  | |  |  | 0.78 |
| Positive (≥10%) | 28 | | 30 | 58 |  |
| Negative (<10%) | 35 | | 34 | 69 |  |
| **Ki67 index** |  | |  |  | <0.001 |
| Low (≤15%) | 39 | | 12 | 51 |  |
| High (＞15%) | 24 | | 52 | 76 |  |
| **Tumor Size** |  | |  |  | <0.001 |
| T1 (≤2cm) | 51 | | 10 | 61 |  |
| T2 (>2cm, ≤5cm) | 10 | | 19 | 29 |  |
| T3-4 (>5cm) | 2 | | 35 | 37 |  |
| **[Lymph node stage](file:///E:\\..\\AppData\\Local\\Youdao\\Dict\\Application\\7.5.2.0\\resultui\\dict\\)** |  | |  |  | <0.001 |
| N0 | 43 | | 17 | 60 |  |
| N1 | 11 | | 10 | 21 |  |
| N2-3 | 9 | | 37 | 46 |  |
| **Recurrence**  **during follow-up** |  | |  |  | <0.001 |
| Yes | 10 | | 29 | 39 |  |
| No | 53 | | 35 | 88 |  |

**Supplementary tables**

| **Table S1. Probes used in current study** | | |
| --- | --- | --- |
| **Gene** | **FISH or ISH (5’ --3’)** | **Pull down (5’ --3’)** |
| circCDYL2 | ATCCTTTCAACCGAGCCCGTTCT | TTGTCTACAATCCTTTCAACCGAGCCCGTTCTCCGCCAGT |
| NC | CCAGTGAATCCGTAATCATG | CCAGTGAATCCGTAATCATG |
| *NC, Negative control* | | |

| **Table S2. The clinical characteristics of circRNA sequencing in HER2^+^ breast cancer patients.** | | | | | | | | |
| --- | --- | --- | --- | --- | --- | --- | --- | --- |
| **Patient number** | **Age** | **ER status**  **(%)** | **PR status**  **(%)** | **Ki67 index (%)** | **Tumor size** | **Lymphatic stage** | **Relapse** | **DFS/follow-up time(months)** |
| R^#^1 | 55 | 0 | 0 | 45 | T2 | N0 | Yes | 24 |
| R2 | 44 | 2 | 0 | 70 | T1 | N1 | Yes | 19 |
| R3 | 29 | 99 | 99 | 1 | T3 | N1 | Yes | 15 |
| R4 | 34 | 80 | 60 | 65 | T2 | N2 | Yes | 14 |
| R5 | 66 | 60 | 0 | 80 | T1 | N1 | Yes | 14 |
| S*^&^*1 | 39 | 2 | 1 | 50 | T2 | N1 | No | 77 |
| S2 | 49 | 99 | 99 | 10 | T1 | N1 | No | 74 |
| S3 | 27 | 0 | 0 | 25 | T2 | N0 | No | 74 |
| S4 | 57 | 90 | 40 | 20 | T1 | N1 | No | 68 |
| S5 | 60 | 75 | 0 | 20 | T1 | N1 | No | 61 |

*# R, trastuzumab-resistant.&S, trastuzumab-sensitive.*

| **Table S3. CircRNAs deep sequencing in HER2^+^ breast cancer tissues.** | | | | | | | | | | | | | | |
| --- | --- | --- | --- | --- | --- | --- | --- | --- | --- | --- | --- | --- | --- | --- |
| **gene_id** | **BC tissues with trasuzumab-resistant**  **(RPM^*^)** | | | | |  | **BC tissues with trastuzumab-sensitive (RPM^*^)** | | | | | ***P* value^#^** | **Fold Change** |  |
|  | **R1** | **R2** | **R3** | **R4** | **R5** |  | **S1** | **S2** | **S3** | **S4** | **S5** |  |  |  |
| CDYL2 | 4.57 | 2.06 | 2.01 | 5.32 | 1.85 |  | 1.01 | 1.79 | 0.00 | 0.00 | 0.00 | 0.013272 | 5.65 |  |
| ANKRD30B | 3.21 | 1.36 | 2.47 | 2.20 | 1.65 |  | 0.76 | 0.16 | 0.00 | 0.73 | 0.32 | 0.001089 | 5.51 |  |
| SPECC1L | 2.94 | 1.79 | 1.76 | 3.12 | 1.47 |  | 0.87 | 1.10 | 0.79 | 0.00 | 0.00 | 0.003541 | 4.02 |  |
| MYO9B | 3.81 | 1.83 | 1.03 | 3.71 | 1.72 |  | 0.22 | 1.56 | 0.69 | 0.56 | 0.00 | 0.019575 | 3.99 |  |
| LYZ | 6.90 | 1.67 | 2.70 | 7.59 | 3.58 |  | 0.09 | 1.15 | 1.79 | 1.15 | 1.60 | 0.024636 | 3.88 |  |
| APOE | 27.96 | 8.00 | 23.21 | 13.30 | 23.43 |  | 3.15 | 3.42 | 6.02 | 6.85 | 5.70 | 0.005467 | 3.81 |  |
| SAT1 | 3.39 | 0.73 | 1.05 | 4.34 | 2.52 |  | 0.03 | 1.27 | 0.58 | 0.73 | 0.55 | 0.037387 | 3.81 |  |
| P4HA1 | 2.56 | 2.02 | 1.54 | 3.03 | 1.45 |  | 0.45 | 0.89 | 0.55 | 0.39 | 0.61 | 0.001196 | 3.66 |  |
| SPATA20 | 1.45 | 4.15 | 1.25 | 1.20 | 2.87 |  | 0.72 | 0.44 | 0.28 | 1.16 | 0.45 | 0.030751 | 3.57 |  |
| RBM5 | 3.73 | 1.25 | 1.61 | 2.95 | 1.69 |  | 0.20 | 1.07 | 0.61 | 0.80 | 0.48 | 0.01113 | 3.55 |  |
| APOE | 34.35 | 10.99 | 30.06 | 16.34 | 32.33 |  | 4.87 | 4.14 | 7.90 | 10.03 | 8.37 | 0.006106 | 3.51 |  |
| CHSY1 | 3.24 | 2.12 | 0.67 | 3.57 | 2.02 |  | 0.28 | 0.00 | 0.81 | 0.67 | 1.64 | 0.02258 | 3.43 |  |
| ADAMTS17 | 6.01 | 2.35 | 1.73 | 2.65 | 3.09 |  | 0.96 | 0.48 | 0.79 | 1.51 | 0.94 | 0.019394 | 3.38 |  |
| PDE4B | 5.24 | 2.13 | 1.64 | 2.05 | 2.98 |  | 0.36 | 1.81 | 1.16 | 0.84 | 0.00 | 0.025118 | 3.37 |  |
| APOE | 37.39 | 13.45 | 34.43 | 19.23 | 36.07 |  | 4.97 | 4.03 | 8.83 | 12.17 | 12.11 | 0.00537 | 3.34 |  |
| NCOR2 | 5.83 | 3.62 | 4.93 | 2.19 | 1.76 |  | 0.51 | 1.56 | 1.08 | 1.81 | 0.80 | 0.014883 | 3.18 |  |
| PPP6R3 | 3.68 | 1.21 | 1.37 | 4.10 | 1.79 |  | 0.13 | 1.55 | 0.60 | 0.62 | 0.94 | 0.033992 | 3.16 |  |
| PSAP | 5.48 | 2.57 | 2.94 | 2.25 | 2.65 |  | 1.74 | 1.15 | 1.20 | 0.52 | 0.52 | 0.009089 | 3.10 |  |
| APOL6 | 3.70 | 1.98 | 1.37 | 1.71 | 1.77 |  | 0.67 | 1.04 | 0.54 | 0.59 | 0.56 | 0.009503 | 3.10 |  |
| GIMAP6 | 3.71 | 1.54 | 0.86 | 3.59 | 1.59 |  | 0.50 | 1.06 | 0.69 | 0.85 | 0.65 | 0.034193 | 3.01 |  |
| **RPM, junction reads per million reads*  *# P value detected by student’s t test*  *R, trastuzumab-resistant patient.&S,trastuzumab-sensitive patient.* | | | | | | | | | | | | | | |

| **Table S4. The clinical characteristics of identified HER2^+^breast cancer patients by qRT-PCR.** | | | | | | | | |
| --- | --- | --- | --- | --- | --- | --- | --- | --- |
| **Patient number** | **Age** | **ER status**  **(%)** | **PR status**  **(%)** | **Ki67 index (%)** | **Tumor size** | **Lymphatic stage** | **Relapse** | **DFS/follow-up time(months)** |
| R1 | 32 | 99 | 99 | 10 | T1 | N0 | Yes | 47 |
| R2 | 45 | 55 | 40 | 35 | T1 | N2 | Yes | 35 |
| R3 | 34 | 85 | 75 | 70 | T1 | N1 | Yes | 33 |
| R4 | 51 | 0 | 0 | 15 | T1 | N0 | Yes | 30 |
| R5 | 49 | 70 | 5 | 30 | T1 | N2 | Yes | 30 |
| R6 | 45 | 3 | 0 | 55 | T2 | N1 | Yes | 30 |
| R7 | 34 | 50 | 60 | 50 | T1 | N0 | Yes | 29 |
| R8 | 40 | 5 | 0 | 60 | T1 | N1 | Yes | 20 |
| R9 | 58 | 2 | 0 | 20 | T1 | N0 | Yes | 19 |
| R10 | 51 | 0 | 0 | 60 | T2 | N2 | Yes | 19 |
| R11 | 38 | 90 | 3 | 15 | T2 | N1 | Yes | 17 |
| R12 | 58 | 1 | 0 | 70 | T1 | N0 | Yes | 16 |
| R13 | 63 | 0 | 1 | 35 | T2 | N1 | Yes | 14 |
| R14 | 48 | 10 | 0 | 45 | T2 | N1 | Yes | 14 |
| R15 | 36 | 5 | 1 | 20 | T2 | N1 | Yes | 12 |
| R16 | 42 | 35 | 0 | 2 | T1 | N3 | Yes | 11 |
| R17 | 33 | 1 | 0 | 90 | T2 | N0 | Yes | 62 |
| R18 | 57 | 0 | 0 | 98 | T2 | N0 | Yes | 60 |
| R19 | 48 | 99 | 5 | 45 | T2 | N3 | Yes | 86 |
| R20 | 60 | 10 | 2 | 25 | T2 | N0 | Yes | 60 |
| R21 | 37 | 99 | 99 | 80 | T1 | N2 | Yes | 63 |
| R22 | 62 | 70 | 0 | 80 | T2 | N2 | Yes | 60 |
| R23 | 36 | 1 | 0 | 20 | T4 | N2 | Yes | 61 |
| R24 | 67 | 0 | 0 | 75 | T2 | N0 | Yes | 63 |
| R25 | 58 | 20 | 0 | 70 | T3 | N0 | Yes | 61 |
| R26 | 54 | 5 | 0 | 5 | T3 | N1 | Yes | 62 |
| R27 | 41 | 0 | 0 | 60 | T1c | N3 | Yes | 86 |
| R28 | 35 | 15 | 0 | 60 | T1c | N2 | Yes | 71 |
| R29 | 52 | 10 | 0 | 65 | T1c | N3 | Yes | 96 |
| R30 | 48 | 99 | 99 | 60 | T3 | N2 | Yes | 67 |
| R31 | 59 | 0 | 0 | 20 | T2 | N2 | Yes | 60 |
| R32 | 49 | 75 | 1 | 50 | T3 | N3 | Yes | 62 |
| R33 | 37 | 1 | 0 | 40 | T3 | N0 | Yes | 13 |
| R34 | 60 | 45 | 0 | 45 | T3 | N0 | Yes | 17 |
| R35 | 58 | 0 | 0 | 35 | T3 | N3 | Yes | 27 |
| R36 | 35 | 3 | 0 | 40 | T3 | N3 | Yes | 17 |
| R37 | 35 | 15 | 35 | 5 | T2 | N3 | Yes | 26 |
| R38 | 32 | 0 | 0 | 30 | T3 | N0 | Yes | 26 |
| R39 | 67 | 95 | 15 | 30 | T3 | N0 | Yes | 4 |
| R40 | 63 | 5 | 2 | 35 | T2 | N3 | Yes | 5 |
| R41 | 38 | 1 | 0 | 25 | T3 | N2 | Yes | 14 |
| R42 | 51 | 0 | 0 | 1 | T2 | N3 | Yes | 13 |
| R43 | 48 | 0 | 0 | 25 | T2 | N2 | Yes | 53 |
| R44 | 61 | 1 | 0 | 20 | T1 | N3 | Yes | 4 |
| R45 | 34 | 80 | 30 | 5 | T4 | N2 | Yes | 4 |
| R46 | 53 | 65 | 0 | 30 | T1 | N0 | Yes | 11 |
| R47 | 56 | 1 | 99 | 30 | T1 | N0 | Yes | 11 |
| R48 | 60 | 0 | 0 | 30 | T1 | N0 | Yes | 21 |
| R49 | 46 | 1 | 0 | 28 | T1 | N0 | Yes | 21 |
| R50 | 35 | 95 | 90 | 25 | T1 | N1 | Yes | 28 |
| R51 | 47 | 60 | 10 | 15 | T1 | N1 | Yes | 58 |
| R52 | 45 | 95 | 95 | 10 | T1 | N0 | Yes | 35 |
| R53 | 57 | 0 | 0 | 30 | T1 | N0 | Yes | 10 |
| R54 | 52 | 2 | 0 | 5 | T2 | N2 | Yes | 39 |
| R55 | 59 | 99 | 99 | 45 | T1 | N3 | Yes | 8 |
| S1 | 41 | 0 | 0 | 80 | T1 | N0 | No | 62 |
| S2 | 38 | 0 | 0 | 70 | T2 | N0 | No | 60 |
| S3 | 48 | 99 | 99 | 60 | T1 | N3 | No | 86 |
| S4 | 56 | 5 | 5 | 55 | T1 | N1 | No | 60 |
| S5 | 52 | 5 | 0 | 55 | T2 | N0 | No | 63 |
| S6 | 40 | 0 | 0 | 50 | T2 | N0 | No | 60 |
| S7 | 53 | 60 | 0 | 40 | T1 | N0 | No | 61 |
| S8 | 58 | 3 | 0 | 40 | T2 | N0 | No | 63 |
| S9 | 55 | 5 | 0 | 30 | T2 | N0 | No | 61 |
| S10 | 51 | 2 | 0 | 25 | T2 | N0 | No | 62 |
| S11 | 52 | 99 | 99 | 20 | T1 | N3 | No | 86 |
| S12 | 31 | 99 | 99 | 20 | T1 | N0 | No | 71 |
| S13 | 30 | 99 | 99 | 20 | T2 | N0 | No | 96 |
| S14 | 61 | 99 | 0 | 20 | T2 | N0 | No | 67 |
| S15 | 44 | 0 | 0 | 15 | T2 | N0 | No | 60 |
| S16 | 50 | 10 | 0 | 10 | T2 | N0 | No | 62 |

*R, trastuzumab-resistant patient.&S,trastuzumab-sensitive patient*

*ER, estrogen receptor*

*PR, progestin receptor*

*DFS. Disease-free survival*

| **Table S5. The clinical characteristics of PDX patient model.** | | | | | | | | |
| --- | --- | --- | --- | --- | --- | --- | --- | --- |
| **Patient number** | **Age** | **ER status**  **(%)** | **PR status**  **(%)** | **Ki67 index (%)** | **Tumor size** | **Lymphatic stage** | **Relapse** | **DFS/follow-up time(months)** |
| ^#^1 | 50 | 80 | 80 | 60 | T2 | N1 | Yes | 8 |
| ^#^2 | 48 | 1 | 1 | 50 | T2 | N0 | Yes | 10 |

| **Table S6. The mass spectrum of proteins after circRNA pull down.** | | | | |
| --- | --- | --- | --- | --- |
| **Gene Symbol** | **Exp. q-value: Combined** | **#Unique Peptides** | **MW [kDa]** | **Reactome Pathways** |
| GRB7 | 0 | 9 | 59.6 | GRB7 events in ERBB2 signaling |
| UGP2 | 0 | 14 | 56.9 | Formation of the active cofactor, UDP-glucuronate |
| GSR | 0 | 7 | 56.2 | Detoxification of Reactive Oxygen Species |
| FAK | 0 | 11 | 120 | Integrin signaling |
| DDX6 | 0 | 5 | 54.4 | mRNA decay by 5' to 3' exoribonuclease |
| SLC2A1 | 0 | 7 | 54 | Lactose synthesis |
| CAP1 | 0 | 16 | 51.9 | Platelet degranulation |
| FKBP4 | 0 | 24 | 51.8 | Attenuation phase |
| EIF5 | 0 | 6 | 49.2 | GTP hydrolysis and joining of the 60S ribosomal subunit |
| DBNL | 0 | 10 | 48.2 | Neutrophil degranulation |

| **Table S7. Different length sequence of circCDYL2 in pull down assay** | | |
| --- | --- | --- |
| **No.** | **Location(~nt)** | **Sequence** |
| Section 1 | 481~592 nt | CACTTTGGTGTGACGTGA…...ACTGGCGGAGAACGGGCTCG |
| Section 2 | 361~592 nt | GACAGGGCCACCAAGAC…...ACTGGCGGAGAACGGGCTCG |
| Section 3 | 241~592 nt | CTGTCCCACAGACCTTCA…...ACTGGCGGAGAACGGGCTCG |
| Section 4 | 121~592 nt | GAGGAGTTTATTGATGAA…...ACTGGCGGAGAACGGGCTCG |
| Section 5 | 1~592 nt | GTTGAAAGGATTGTAGAC…...ACTGGCGGAGAACGGGCTCG |

| **Table S8. Sequence of siRNA or shRNA used in current study** | | | |
| --- | --- | --- | --- |
| **Gene** |  | **Sense (5’ --3’)** | **Antisense (5’ --3’)** |
| circCDYL2 | si/sh-1 | GAGAACGGGCUCGGUUGAAdTdT | UUCAACCGAGCCCGUUCUCdTdT |
|  | si/sh-2 | GCUCGGUUGAAAGGAUUGUdTdT | ACAAUCCUUUCAACCGAGCdTdT |
| HER2 | si-1 | GGAGACCCGCUGAACAAUAdTdT | UAUUGUUCAGCGGGUCUCCdTdT |
|  | si-2 | CAGACACGUUUGAGUCCAUdTdT | AUGGACUCAAACGUGUCUGdTdT |
| GRB7 | si-1 | GGAAAAACUUCGCCAAGUAdTdT | UACUUGGCGAAGUUUUUCCdTdT |
|  | si-2 | CUGGGCGUGUCAUUGAGAAdTdT | UUCUCAAUGACACGCCCAGdTdT |
| FAK | si | UAAUACUCGCUCCAUUGCACCdTdT | GGUGCAAUGGAGCGAGUAUUAdTdT |
| Pin1 | si | GCAGCAGUGGUGGCAAAAAdTdT | UUUUUGCCACCACUGCUGCdTdT |

| **Table S9. Sequence of overexpression plasmid used in current study** | | |
| --- | --- | --- |
| **Gene** |  | **Sense (5’ --3’)** |
| circCDYL2 | overexpress/p-circ | GTTGAAAGGATTGTAGACAAGAGGA......GCTACACTGGCGGAGAACGGGCTCG |
| GRB7 | overexpress | ATGGAGCTGGATCTGTCTCCACCTC......TTGCTGCACGCGGGTGGCCCTCTGA |
| FAK | overexpress | ATGTTGTTGGAACTGGCAGGCCAAG......GCTTGGGCAGACGAGACCACACTGA |
| Pin1 | overexpress | ATGGCGGACGAGGAGAAGCTGCCGC......CCACATCATCCTCCGCACTGAGTGA |

| **Table S10. Primers used in current study** | | |
| --- | --- | --- |
| **Gene** | **Forward primer (5’ --3’)** | **Reverse primer (5’ --3’)** |
| circCDYL2 | CCTGGCTTGGATTTGAATGAT | TTCCATCGGATAAGATACTCCC |
| linear CDYL2 | GGCCGGTTGTCCAGGGA | GAGGCGGATGGTGGCGTAG |
| HER2 | TGCAGGGAAACCTGGAACTC | ACAGGGGTGGTATTGTTCAG |
| GRB7 | GCTGGGCGTGTCATTGAG | GAGTTGGGTGCGGTGGAT |
| FAK | GCTTACCTTGACCCCAACTTG | ACGTTCCATACCAGTACCCAG |
| β-actin | TCATGAAGTGTGACGTGGACATC | CAGGAGGAGCAATGATCTTGATCT |
| GAPDH | AGGTGAAGGTCGGAGTCAAC | CGCTCCTGGAAGATGGTGAT |
